# Thermoelectric properties of heavy fermion CeRhIn_5_ using density functional theory combined with semiclassical Boltzmann theory†

**DOI:** 10.1039/c9ra07859b

**Published:** 2019-11-06

**Authors:** M. Yazdani-Kachoei, S. Jalali-Asadabadi

**Affiliations:** Department of Physics, Faculty of Sciences, University of Isfahan (UI) Hezar Gerib Avenue Isfahan 81746-73441 Iran saeid.jalali.asadabadi@gmail.com sjalali@sci.ui.ac.ir +98 31 37934800 +98 31 37932435

## Abstract

Experimental evidences show that Ce-based compounds can be good candidates for thermoelectric applications due to their high thermoelectric efficiencies at low temperatures. However, thermoelectric properties have been studied less than the other properties for CeRhIn_5_, a technologically and fundamentally important compound. Thus, we comprehensively investigate the thermoelectric properties, including the Seebeck coefficient, electrical conductivity, electronic part of thermal conductivity, power factor and electronic figure of merit, by a combination of quantum mechanical density functional and semiclassical Boltzmann theories, including relativistic spin–orbit interactions using different exchange–correlation functionals at temperatures *T* ≤ 300 K for CeRhIn_5_ along its *a* and *c* crystalline axes. The temperature dependences of the thermoelectric quantities are investigated. Our results reveal a better Seebeck coefficient, electrical conductivity, power factor and thermoelectric efficiency at *T* ≪ 300, in agreement with various other Ce-based compounds, when a high degree of localization is considered for the 4f-Ce electrons. The Seebeck coefficient, power factor and thermoelectric efficiency are made more efficient near room temperature by decreasing the degree of localization for 4f-Ce electrons. Our results also show that the thermoelectric efficiency along the *a* crystalline axis is slightly better than that of the *c* axis. We also investigate the effects of hydrostatic pressure on the thermoelectric properties of the compound at low and high temperatures. The results show that the effects of imposing pressure strongly depend on the degree of localization considered for 4f-Ce electrons.

## Abstract 2

Consistent with experimental data, theoretical thermoelectric results calculated by our developed strategy show that CeRhIn_5_ is a good candidate for thermoelectric cooling applications due to its high thermoelectric efficiency at low temperatures.

## Graphical abstract

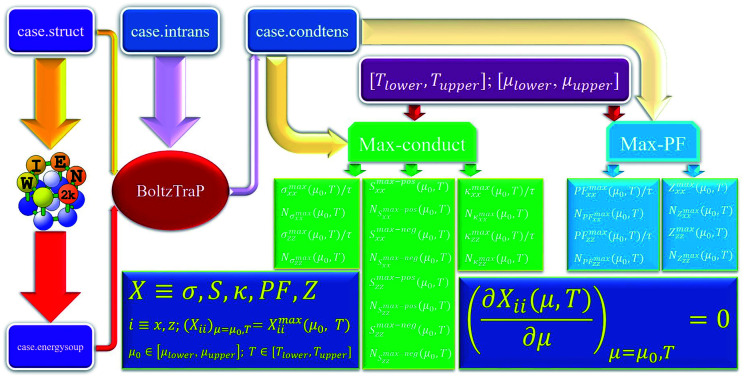

## 1 Introduction

The Peltier cooling mechanism using thermoelectric (TE) materials has attracted considerable attention as a replacement for common refrigeration methods.^1–8^ In contrast to common cooling techniques, the Peltier mechanism can be more useful in small scale and site-specific applications. However, the applications of this method are limited by the low efficiency of TE materials. Therefore, many researches have been performed to explore new efficient TE materials and improve the efficiency of the available TE materials as renewable energy resources.^9–18^ The thermoelectric efficiency of a material is characterized by its figure of merit (*ZT*) parameter, where *Z* is 
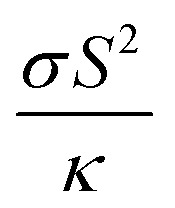
, *σ* is the electrical conductivity, *S* is the Seebeck coefficient and *κ* is the thermal conductivity of the compound. Usually, the *ZT* value is equal to or more than unity for an efficient thermoelectric material.^19^ Rare earths, and specifically cerium-based compounds, compose a class of strongly correlated materials that exhibit good thermoelectric properties with large *Z* values at low temperatures (*T* ≤ 300 K).^20–29^ Among them, CeRhIn_5_ has attracted considerable attention due to its fascinating properties. So far the electronic,^30–34^ magnetic^32,35–38^ and optical properties^39^ of this compound have been extensively studied both theoretically and experimentally. CeRhIn_5_ is a well-known heavy-fermion antiferromagnet that becomes a bulk heavy-fermion superconductor at higher pressures and very low temperatures, as would be seen in the (*P*, *T*) phase diagram of this compound in zero field.^40^ In this work, the range of temperatures is much higher, *i.e.*, up to about *T* = 300 K, than the superconductivity dome. Comprehensive experimental researches have been performed for CeRhIn_5_ in the last decade, including a detailed study of the heat and charge transport properties.^41–43^ Despite strong experimental evidences of high thermoelectric efficiency for the other Ce-based compounds, the thermoelectric efficiency of CeRhIn_5_ has not been theoretically or experimentally investigated yet. All this motivated us to theoretically study the thermoelectric efficiency of CeRhIn_5_ compound using a combination of quantum mechanical density functional theory (DFT)^44,45^ and semiclassical Boltzmann theories.^46,47^ Thus, one of the important aims of this study is to find the temperature range in which the maximum thermoelectric efficiency can be observed. Here, we calculate the Seebeck coefficient (*S*), electrical conductivity (*σ*), electronic part of thermal conductivity (*κ*_e_) and numerator of the *Z* ratio, *i.e.*, power factor (PF = *σS*^2^), along both *a* and *c* crystalline axes at zero pressure. So far, in most of the theoretical Boltzmann based investigations of thermoelectric properties, the aforementioned characters, *i.e.*, *S*, *σ*, *κ*_e_, PF, have been calculated as functions of chemical potential at some fixed temperature or *vice versa*; as functions of temperature at some fixed chemical potential. In the present study, however, we calculate these characters as functions of temperature and find their maximum values to discuss in which temperature range the best thermoelectric efficiency can be achieved.

Our results show that the maximum value for the Seebeck coefficient occurs at low temperatures for CeRhIn_5_, consistent with previous experimental reports for other Ce-based compounds,^13,20,21,24,48^ when the 4f-Ce electrons lie in the high localized regime. Furthermore, based on our results the *Z*_e_ value of our studied case in lower temperatures is more than that of higher temperatures.

Experimental results^24,25,49^ have shown that applying pressure can affect the thermoelectric efficiency of the other rare earth based compounds. In addition, experimental measurements^41,43^ have shown that imposing pressure can affect the electrical conductivity and thereby the thermoelectric efficiency of CeRhIn_5_. Moreover, Shishido *et al.*^50,51^ have experimentally verified that the degree of localization of 4f-electrons in CeRhIn_5_ is decreased by imposing pressure. Thus, these experimental evidences motivated us to investigate the effects of pressure as well as the degree of localization of 4f-Ce electrons on the thermoelectric properties and thermoelectric efficiency of CeRhIn_5_. For this purpose, we have performed our calculations in different volumes using various exchange–correlation functionals (XCFs) with different degrees of localization for 4f-Ce electrons, including LDA+U^52^ and hybrid^53,54^ approaches as well as PBE-GGA. Our results show that the Seebeck coefficient of CeRhIn_5_, its power factor and *Z*_e_ value increase as the degree of localization decreases at high temperatures. This shows that the Seebeck coefficient, power factor and *Z*_e_ value are made more efficient near room temperature by decreasing the degree of localization of 4f-Ce electrons. Our results also reveal that the effect of hydrostatic pressure on the thermoelectric parameters strongly depends on the considered degree of localization for 4f-Ce electrons, in accordance with our previous work.^55^ The electronic structures of CeRhIn_5_, including its density of states (DOS) and band structure are also investigated in this study.

## 2 Details of calculations

CeRhIn_5_ crystallizes in *P*4/*mmm* space group number 123.^56–58^ The chemical structure of this material is shown in Fig. 1 of the ESI.† Our calculations are performed using experimental lattice parameters *a* = 4.656 (Å) and *c* = 7.542 (Å).^57,58^ The electronic calculations in the present work are performed in the framework of density functional theory (DFT)^44,45^ using the WIEN2k code through the full potential APW+lo method^59^ in the presence of spin–orbit coupling (SOC) . The GGA+U with *U*_eff_ = 5.5 eV, hybrid B3PW91 with *α* = 0.30 and PBE-GGA approaches are used to describe the exchange–correlation term. A grid of 18 × 18 × 11 for CeRhIn_5_ in the scheme of Monkhorst–Pack is selected for the mesh of *k* points for the electronic structure calculations. The cutoff parameters *K*_max_, *l*_max_ and *G*_max_ are set to 8(*R*_MT_)^−1^, 10 and 12 bohr^−1^, respectively. The muffin-tin radii (*R*_MT_) are chosen to be 2.8 a.u. for Ce and 2.2 a.u. for In and Rh. We performed the full-relaxation to reduce the total forces on all atoms down to values smaller than 0.5 mRy bohr^−1^. In addition to the volume corresponding to the experimental volume (first volume), the calculations have been performed for two other volumes, where the second volume is 2% smaller and the third volume is 5% smaller than the first volume. From now on, in the rest of this paper and its corresponding ESI, these three cases are called “the first volume”, “the second volume” and “the third volume”.

The transport properties are calculated by BoltzTraP code^61^ which is based on the Boltzmann theory.^46,47^ Since this code needs a very high *k*-mesh, a denser mesh of 150 000 *k*-points is considered for the thermoelectric calculations. The Seebeck coefficient, electrical conductivity and the electronic part of the thermal conductivity tensors are calculated by the following formulas:^61,62^1
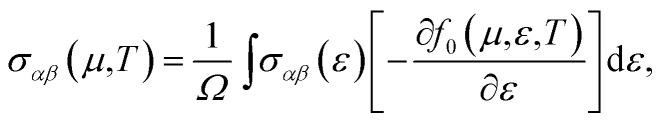
2
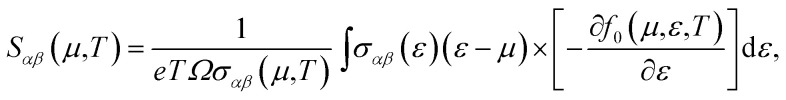
3
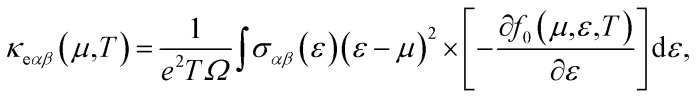
where *μ* is the chemical potential, *Ω* is the volume of unit cell, *α* and *β* are the tensor indices, *f*_0_ is the Fermi–Dirac distribution function, *e* is the charge carrier and4
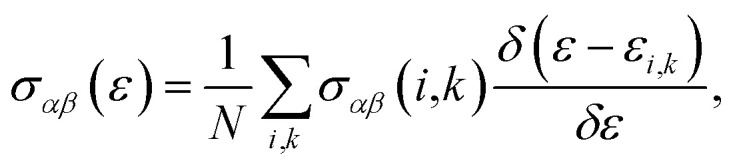
while

5 *σ*_*αβ*_(*i*,*k*) = *e*^2^*τ*_*i*,*k*_*υ*_*α*_(*i*,*k*)*υ*_*β*_(*i*,*k*),

where *N* is the number of *k* points, *τ* is the relaxation time and *υ*_*α*_(*i*,*k*) is the component of group velocities. It is noticeable that the thermal conductivity and electrical conductivity are calculated with respect to the relaxation time here. According to ref. 63, the relaxation time approximation is valid for high doping levels >1 × 10^18^ carriers per cm^3^. In this work, the range of doping levels is limited to vary from −10^21^ to 10^21^ carriers per cm^3^, which is realized in the experiment and consistent with previous theoretical results in other cases.^64^ The limitation of the doping levels is equivalent to the limitation of the chemical potential.^65,66^ In our case, the limited doping level [−10^21^, 10^21^ carriers per cm^3^] interval which is equivalent to [−1.6 to 1.6 carriers per uc] can be corresponded to a chemical potential interval of [−0.7, 0.7 eV] around the Fermi level (*E*_F_). The key input parameters for BoltzTraP calculations^61^ are set as follows: delta = 0.0001 Ry, cut-off energy around the Fermi level or ecut = 0.5, energy range of chemical potential or efcut = 0.4 Ry, maximum considered temperature of *T*_max_ = 300 K, temperature grid = 1 K and lpfac = 30.

In many previous works, the thermoelectric parameters have been studied at fixed temperature *versus* chemical potential (*μ*) or at fixed *μ versus* temperature (*T*). However, as eqn (1)–(3) in the manuscript show, the elements of electrical conductivity (*σ*_*αβ*_(*μ*,*T*)), Seebeck coefficient (*S*_*αβ*_(*μ*,*T*)) and thermal conductivity (*κ*_*αβ*_(*μ*,*T*)) tensors are the functions of two parameters, *i.e. μ* and *T*. The BoltzTraP code calculates these parameters *versus T* and *μ*. In fact, BoltzTraP changes the value for *μ* step by step and calculates *σ*_*αβ*_(*μ*,*T*), *S*_*αβ*_(*μ*,*T*) and *κ*_*αβ*_(*μ*,*T*) at fixed *μ versus* temperature at each step. Thus, a change of the *μ* or *T* values can change *σ*_*αβ*_(*μ*,*T*), *S*_*αβ*_(*μ*,*T*), *κ*_*αβ*_(*μ*,*T*). Therefore, to optimize the thermoelectric parameters, *μ* and *T* should be considered simultaneously. For this, we follow a new strategy to analyze the outputs of BoltzTraP code. In our strategy, we change the temperature step by step (1 K in our calculations) and find the maximum of thermoelectric parameters for fixed temperature at each step from the outputs of BoltzTraP code, *i.e.*, case.condtens and the value of *μ* or doping level which leads to this maximum value. We encounter too many numbers in the case.condtens. Therefore, to find the maximum values of *σ*_*αβ*_(*μ*,*T*), *S*_*αβ*_(*μ*,*T*) and *κ*_*αβ*_(*μ*,*T*) at fixed *μ*, we use a simple program, max-conduct. To calculate the maximum values of power factor (PF = *σS*^2^) and electronic figure of merit 
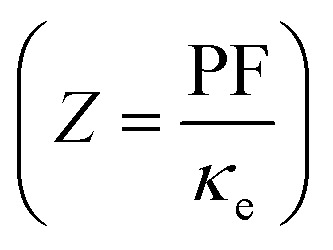
, we use another program, *i.e.* max-PF. This program calculates the diagonal elements of PF = *σS*^2^ and 
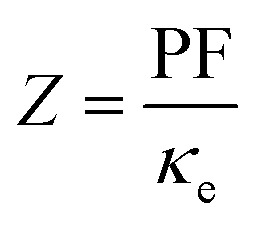
*versus μ* and *T*, then finds the maximum values of these parameters the same as *S*, *σ* and *κ*.

## 3 Electronic structure

Thermoelectric properties of materials can be related to their corresponding electronic structures.^19,67–70^ For metals or degenerate semiconductors, the Seebeck coefficient can be written as:^19,67,71^6
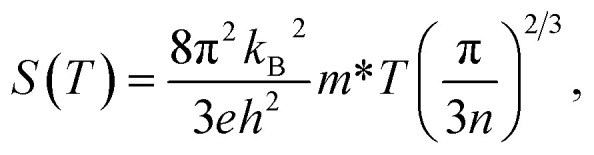
where *n* is the carrier concentration and m* is the effective mass of the carrier which depends on the electronic structure of the system; the higher the DOS(*E*_F_), the larger the m*. *k*_B_ and *h* are the Boltzmann and Planck constants, respectively, and *e* is the electron charge. Furthermore, from the Mott relation we have:^69,72^7
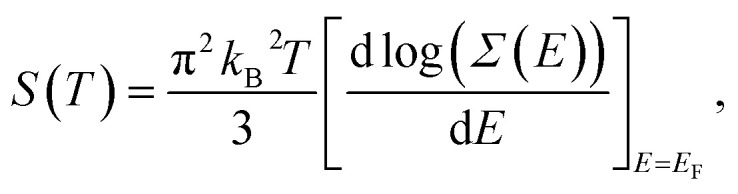
where *Σ*(*E*) = *N*(*E*)*v*^2^(*E*)*τ*(*E*) is the transport parameter. Here, *N*(*E*), *v*(*E*) and *τ*(*E*) are the DOS(*E*), Fermi velocity and scattering time. The electrical conductivity (*σ*) and electrical resistivity (*ρ*) are given by:^19,67^

8 *σ* = 1/*ρ* = *neμ*,

where *μ* is the carrier mobility which can be related to the effective masses of the carriers. This relationship also depends on the electronic structure of the system. Furthermore, the thermal conductivity is related to the electronic structure through the electrical conductivity and Wiedemann–Franz law.

All the above evidences explicitly confirm that the thermoelectric parameters depend on the electronic structures of the materials. Thus, the electronic structure plays a key role and a discussion in this respect can give physical insight into the thermoelectric properties of the system in question. Before discussing the electronic structure results, however, it is important to consider and keep in mind the following three points. The first point is that to perform accurate electronic structure calculations, it is essential to be aware of the degree of localization of the system for selecting an appropriate functional to satisfactorily deal with the exchange–correlation term. The following evidences may assist with the first point. Previous dHvA measurements show that 4f-Ce electrons in CeRhIn_5_ are localized at zero pressure.^50,51^ Experimental measurements^73^ performed at zero pressure by Fujimori *et al.* also confirm that the 4f-Ce electrons in CeRhIn_5_ have localized character. Furthermore, the dynamical mean field theory calculations^30^ demonstrate that 4f-Ce electrons of CeRhIn_5_ are more localized than those of CeCoIn_5_ and CeIrIn_5_ at zero pressure. The latter cases (CeCoIn_5_ and CeIrIn_5_) themselves are also demonstrated by ARPES measurements to be localized systems.^74,75^ All these theoretical and experimental evidences clearly show that the 4f-Ce electrons are localized in CeRhIn_5_ at zero pressure. The second point which should be considered for producing accurate electronic structures concerns the validity of the functional used for the exchange–correlation term. It is well-known that the standard GGA often fails to reproduce the correct electronic structures for strongly correlated f-electron systems. For highly correlated systems, the thermoelectric properties, especially the thermopower S, can be strongly influenced by the location of the narrow DOSs with respect to the Fermi level. Therefore, in this work, we use the band-correlated GGA+U and hybrid B3PW91 approaches to investigate the electronic structures of CeRhIn_5_. The third point, which is also crucial for an accurate prediction of the electronic structure, concerns the pressure dependence of the localization degree. Although the 4f-Ce electrons are localized in CeRhIn_5_ at zero pressure, the dHvA measurements^50,51^ show that the localization degree of 4f-Ce electrons in CeRhIn_5_ is pressure dependent. This implies that the localization degree of these electrons can be reduced by imposing pressure. Furthermore, we discussed in our recent work,^55^ in agreement with previous works,^76^ that the exchange–correlation energy of Ce-based compounds could not be satisfactorily described only by a single functional for every pressure. Therefore, for a specific pressure range an appropriate functional must be selected; band-correlated (band-like) functionals are more appropriate for low (high) pressures.^55^ Thus, following this strategy, in addition to the band-correlated GGA+U and hybrid B3PW91 approaches, the band-like PBE-GGA functional is also used for the electronic structure investigation.

**Fig. 1:**
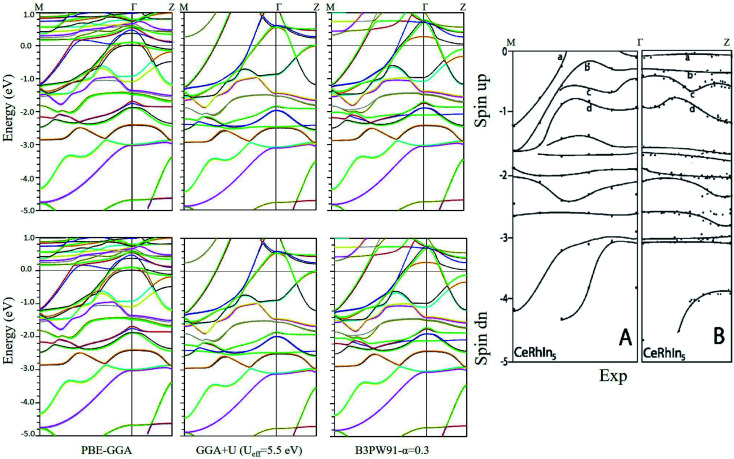
Spin up and spin down band structures calculated by PBE-GGA, GGA+U with *U*_eff_ = 5.5 eV and hybrid B3PW91 with *α* = 0.3 for CeRhIn_5_ using the experimental volume. In the right figure, the ARPES experimental result^60^ is also shown for comparison.

**Fig. 2:**
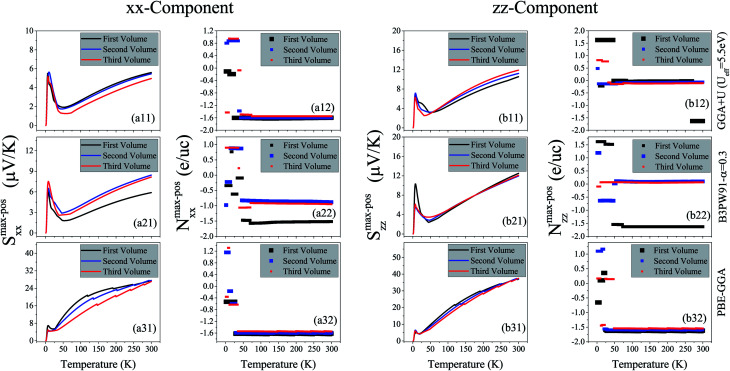
Maximum positive or hole-like of spin up Seebeck coefficient *versus* temperature calculated by GGA+U with *U*_eff_ = 5.5 eV, B3PW91 with *α* = 0.30 and PBE-GGA for the three different volumes of CeRhIn_5_. The first volume corresponds to the experimental volume at zero pressure. The second and third volumes are 2% and 5% smaller than the first volume, respectively. The (a*i*1) panels display the *xx* components, *i.e.*, *S*^max-pos^_*xx*_, while the (b*i*1) panels display the *zz* components, *i.e.*, *S*^max-pos^_*zz*_. Doping levels related to the *S*^max-pos^_*xx*_ and *S*^max-pos^_*zz*_ are displayed in the (a*i*2) and (b*i*2) panels, respectively. The *i*-index varies from 1 to 3.

Now, we can discuss the electronic structure of the system by considering the above points. To this end, we have calculated the spin up and spin down band structures of CeRhIn_5_ compound using PBE-GGA, PBE-GGA+U with *U*_eff_ = 5.5 eV and hybrid B3PW91 with *α* = 0.3 for the three volumes introduced in Section 2. Here, the band structures are shown only for the first volume (which corresponds to the experimental volume) together with the available experimental results^60^ for comparison to validate our calculations in Fig. 1. The complete results including band characters are presented in detail in Fig. 2 and 3 of the ESI.† The comparison confirms that our calculated band structures are consistent with the experimental results.

We have found that the pressure cannot considerably change the band structures of the system for spins up and down using PBE-GGA. We have also observed that the number of bands crossing the Fermi level is 5 and not changed by pressure using PBE-GGA. To validate these observations, the maximum and minimum energies and bandwidths, as well as occupation numbers, are extracted from the calculated band structures and tabulated in Table 1. The results show that the PBE-GGA bands data can be only slightly changed by pressure. Although the widths of the 5 PBE-GGA bands are increased by pressure (due to the small decrease of the minimum energies and the small increase of their maximum energies), these increments are not very considerable. We noticed that in contrast to the pressure, however, the degree of localization could more considerably affect the band structures. If instead of the band-like PBE-GGA, the band-correlated PBE-GGA+U and B3PW91 functionals are used, more bands (6 bands) cross the Fermi level. The latter number of band crossing (6 bands) is different from that obtained using the band-like PBE-GGA (5 bands). This difference confirms a more significant effect of the localization degree than pressure in complete agreement with the result reported for CeIn_3_ in our recent work.^55^ This result can be reconfirmed if we consider the fact that the degree of localization predicted by PBE-GGA is lower than those predicted by GGA+U and B3PW91. Keeping the latter fact in mind, we see in Table 1 that, in contrast to pressure, the bands data are substantially affected by the degree of 4f localization. The results show that the minimum energy (*E*_min_) decreases as the degree of localization increases and inversely the results show that the maximum energy (*E*_max_) increases as the degree of localization increases. Therefore, the bandwidths increase for the five bands, but this time, the increments of the bandwidths are remarkable and should be considered. The difference between the effect of pressure and localization degree can be also seen in the band structures presented in the ESI,† if we follow the effects of localization degree and pressure on the distributions of the 4f-Ce states and their locations with respect to the Fermi level. These results are in good agreement with previous works.^32^

**Table 1:** Maximum energy (*E*_max_), minimum energy (*E*_min_), bandwidth (width) and occupation number (occup) of bands crossing the Fermi level within various functionals in various volumes of CeRhIn_5_. The first volume corresponds to the experimental volume at zero pressure, the second and third volumes are 2% and 5% smaller than the first volume, respectively. *E*_max_ and *E*_min_ are measured with respect to the Fermi level so that positive energies are located above the Fermi level while negative energies are located below the Fermi level.

XCF	Band	First volume				Second volume				Third volume			
		*E*_min_ (eV)	*E*_max_ (eV)	Width (eV)	Occup	*E*_min_ (eV)	*E*_max_ (eV)	Width (eV)	Occup	*E*_min_ (eV)	*E*_max_ (eV)	Width (eV)	Occup
PBE-GGA	γ1	−1.3783	0.0729	1.4512	0.9728	−1.4055	0.0753	1.4808	0.9727	−1.4462	0.0791	1.5253	0.9730
	γ2	−1.2030	0.0940	1.2970	0.9269	−1.2328	0.0968	1.3296	0.9247	−1.2783	0.1021	1.3804	0.9211
	γ3	−1.1729	0.3588	1.5317	0.602	−1.2010	0.3579	1.5589	0.6015	−1.2445	0.3537	1.5983	0.6001
	γ4	−1.0897	0.3931	1.4828	0.2954	−1.1154	0.3916	1.5070	0.2948	−1.1539	0.3891	1.5430	0.2944
	γ5	−1.0506	0.4999	1.5505	0.2033	−1.0747	0.4939	1.5686	0.2068	−1.1114	0.4832	1.5947	0.2119
GGA+U (*U*_eff_ = 5.5 eV)	γ1	−1.5310	0.5243	2.0553	0.9186	−1.5491	0.5341	2.0832	0.9154	−1.5754	0.5479	2.1233	0.9116
	γ2	−1.5050	0.5513	2.0563	0.9060	−1.5229	0.5584	2.0812	0.9037	−1.5450	0.5733	2.1183	0.8982
	γ3	−1.3006	1.1148	2.4155	0.3807	−1.3278	1.1328	2.4606	0.3821	−1.3719	1.1543	2.5263	0.3848
	γ4	−1.2836	1.1211	2.4047	0.3747	−1.3113	1.1350	2.4463	0.3765	−1.3526	1.1556	2.5082	0.3789
	γ5	−1.1880	1.5800	2.7680	0.2143	−1.2106	1.6159	2.8265	0.2151	−1.2501	1.6610	2.9112	0.2175
	γ6	−1.1670	1.5914	2.7584	0.2057	−1.1902	1.6314	2.8216	0.2071	−1.2255	1.6943	2.9198	0.2089
B3PW91-*α* = 0.30	γ1	−1.5011	0.4869	1.9880	0.9267	−1.4417	0.2728	1.7145	0.9509	−1.4703	0.3187	1.7890	0.9463
	γ2	−1.4829	0.5660	2.0489	0.9014	−1.4185	0.5566	1.9750	0.9006	−1.4463	0.5589	2.0052	0.9005
	γ3	−1.2863	0.9585	2.2448	0.3821	−1.2466	0.6066	1.8533	0.3952	−1.2786	0.6362	1.9148	0.3913
	γ4	−1.2731	1.0699	2.3430	0.3725	−1.2262	0.8002	2.0264	0.3598	−1.2573	0.8453	2.1025	0.3633
	γ5	−1.1719	1.3956	2.5675	0.2097	−1.1208	0.9026	2.0234	0.2074	−1.1495	0.9265	2.0760	0.2096
	γ6	−1.1580	1.4800	2.6380	0.2076	−1.0938	0.9960	2.0899	0.1859	−1.1218	1.0333	2.1551	0.1890

The above results on the difference between the effects of localization degree and pressure are also supported and reconfirmed by the total and partial up and down DOSs calculated by PBE-GGA, GGA+U and B3PW91 for the three considered volumes of the compound. The DOSs are presented in Fig. 4 and 5 of the ESI,† and here we only quantitatively present the values of the total DOS^tot^(*E*_F_) in Table 2, since the negligible effects of pressure can be straightforwardly seen in the total DOS^tot^(*E*_F_). As clearly seen in Table 2, the DOS^tot^(*E*_F_) using PBE-GGA only very slightly decreases as pressure increases. A similar trend can be seen in this table using GGA+U and hybrid B3PW91 approaches. However, the DOS^tot^(*E*_F_) is much more considerably affected by GGA+U and B3PW91 than PBE-GGA. This theoretical investigation shows that the effect of the degree of localization is more considerable than the weak effect of pressure.

**Table 2:** Total up and down DOSs(*E*_F_) for CeRhIn_5_ using various XCFs for three different volumes of CeRhIn_5_. The first volume corresponds to the experimental volume at zero pressure. The second and third volumes are 2% and 5% smaller than the first volume, respectively.

XCF	Spin	First volume	Second volume	Third volume
PBE-GGA	Up	5.63	5.52	5.34
	Down	1.68	1.65	1.63
GGA+U (*U*_eff_ = 5.5 eV)	Up	0.99	0.97	0.96
	Down	1.04	1.03	1.01
B3PW91−*α* = 0.30	Up	1.63	1.54	1.36
	Down	1.07	1.04	1.02

Despite all the above discussed theoretical differences between the effects of pressure and localization degree, we should note that in experiment, however, the effect of the localization degree is tightly related to the effect of pressure. This is so because the degree of localization is itself changed by pressure for strongly correlated systems in nature.^55,76^ So why is pressure not as effective as localization degree in our above theoretical discussion? Do the theoretical results contradict the experimental results? The answer is that when we apply pressure experimentally, the degree of localization is also naturally changed, but the degree of localization theoretically depends on the functional used. Therefore, when we apply pressure theoretically the degree of localization is not remarkably changed automatically, because in the current available DFT approaches the degree of localization should be applied manually by selecting an appropriate functional and tuning by hand its parameter such as U parameter in GGA+U or *α* parameter in B3PW91 for the exchange–correlation term.^55^ Thus, the theoretical results can be consistent with the experimental results, if a proper degree of localization is considered. This reconfirms our recent report on the pressure dependency of localization degree in CeIn_3_.^55^ This point will be considered in the subsequent sections.

## 4 Seebeck coefficient

**Fig. 3:**
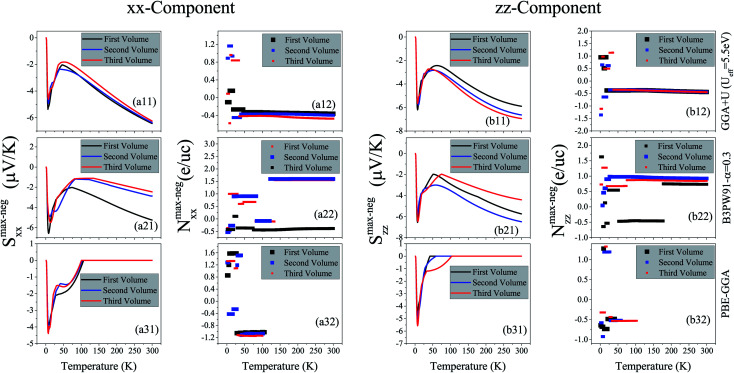
Maximum negative or electron-like of spin up Seebeck coefficient *versus* temperature calculated by GGA+U with *U*_*e*ff_ = 5.5 eV, B3PW91 with *α* = 0.30 and PBE-GGA for the three different volumes of CeRhIn_5_. The first volume corresponds to the experimental volume at zero pressure. The second and third volumes are 2% and 5% smaller than the first volume, respectively. The (a*i*1) panels display the *xx* components, *i.e.*, *S*^max-neg^_*xx*_, while the (b*i*1) panels display the *zz* components, *i.e.*, *S*^max-neg^_*zz*_. Doping levels related to the *S*^max-neg^_*xx*_ and *S*^max-neg^_*zz*_ are displayed in the (a*i*2) and (b*i*2) panels, respectively. The *i*-index varies from 1 to 3.

Materials efficiency in thermoelectricity is evaluated by 
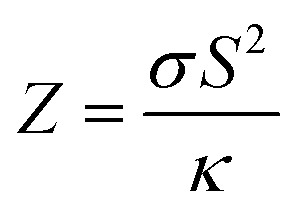
, where *S* is the Seebeck coefficient, *σ* is the electrical conductivity and *κ* is the thermal conductivity. Therefore, the thermoelectric efficiency can be improved by increasing the Seebeck coefficient and electrical conductivity as well as decreasing the thermal conductivity. Seebeck coefficient, as expressed in eqn (2), is a 9-components tensor. However, for our case the three diagonal components, *i.e.*, *S*_*ii*_; (*i* = *x*, *y*, *z*), are much (an order of magnitude) larger than the off-diagonal components, *viz*., *S*_*ij*_ ≪ *S*_*ii*_; (*i*≠*j*). Furthermore, the tetragonal crystal structure requires that *S*_*xx*_ = *S*_*yy*_. Thus, we only investigate the remaining two components of the Seebeck coefficient, *S*_*xx*_ and *S*_*zz*_. The maximum positive *xx* and *zz* components of the Seebeck coefficient, *S*^max-pos^_*xx*_ and *S*^max-pos^_*zz*_, as indicators of hole-like Seebeck coefficient components are calculated as functions of temperature for the three aforementioned volumes of CeRhIn_5_ using PBE-GGA, PBE-GGA+U with *U*_eff_ = 5.5 eV and B3PW91 with *α* = 0.30, as shown in Fig. 2(a*i*1) and (b*i*1) (*i* = 1 to 3) for spin up, respectively. Furthermore, the maximum negative *xx* and *zz* components of the Seebeck coefficient, *S*^max-neg^_*xx*_ and *S*^max-neg^_*zz*_, as indicators of electron-like Seebeck coefficient components are also calculated as functions of temperature for the three aforementioned volumes of CeRhIn_5_ using the PBE-GGA, PBE-GGA+U with *U*_eff_ = 5.5 eV and B3PW91 with *α* = 0.30, as shown in Fig. 3(a*i*1) and (b*i*1) for spin up, respectively. Our results show that the results for spin down are very similar to spin up, so we only discuss the spin up results. One of the important aims of this study is finding the temperature range in which the maximum Seebeck coefficient can be observed. Let us start the Seebeck coefficient discussion with the results of the high localized GGA+U and B3PW91 functionals. A maximum value of about 5.5 μV K^−1^ (6.7 μV K^−1^) can be seen around *T* ≃ 7 K for spin up of *S*^max-pos^_*xx*_ (*S*^max-pos^_*zz*_) in the first volume using the GGA+U functional, as shown in Fig. 2(a11) (Fig. 2(b11)). Likewise, a maximum value of 6 μV K^−1^ (10 μV K^−1^) is predicted by B3PW91 for *S*^max-pos^_*xx*_ (*S*^max-pos^_*zz*_), as shown in Fig. 2(a21) (Fig. 2(b21)). The maximum values predicted by the B3PW91 functional are larger than those predicted by GGA+U. This is in agreement with the direct relation between DOS and Seebeck coefficient (eqn (7)), because B3PW91 DOS(*E*_F_) is higher than GGA+U DOS(*E*_F_), as shown in (Table 2). After *T* ≃ 7 K, the *S*^max-pos^_*xx*_ and *S*^max-pos^_*zz*_ values monotonically started to decrease up to *T* ≃ 50 K in the first volume as predicted by both the B3PW91 and GGA+U approaches. They then increase as temperature increases up to *T* = 300 K, see Fig. 2(a11) and (b11) for GGA+U as well as Fig. 2(a21) and (b21) for B3PW91. The above discussed behavior, *i.e.*, showing a maximum value at low temperatures (*T* ≪ 300 K), was reported experimentally for the Seebeck coefficients of CeIrIn_5_^24^ and CeRhIn,^48^ in agreement with our theoretical predictions. The crystal structure of the CeIrIn_5_ is identical to the CeRhIn_5_; they are isostructural compounds. Furthermore, the constituent elements of CeRhIn are the same as CeRhIn_5_. Moreover, consistent with our results, the same behavior of the Seebeck coefficient was previously reported experimentally not only for the other Ce-based compounds, but also for the other rare earth-based metals.^27,29,77,78^ For the *xx* component of the electron-like Seebeck coefficient, *S*^max-neg^_*xx*_, the GGA+U [B3PW91] functional predicts a local [global] minimum (negative maximum) value of −5.5 μV K^−1^ [−6.6 μV K^−1^] around *T* = 7 K in the first volume, and then this value decreases as temperature increases up to *T* ≃ 50 K [*T* ≃ 70 K]. After *T* ≃ 50 K [*T* ≃ 70 K], the *S*^max-neg^_*xx*_ increases by increase of temperature up to room temperature within GGA+U [B3PW91], as shown in Fig. 3(a11) [Fig. 3(a21)]. The behavior of *S*^max-neg^_*zz*_ within the GGA+U functional is very similar to the *S*^max-neg^_*xx*_ behavior, as shown when comparing the (b11) panel with the (a11) panel of Fig. 3. Similar behavior is also predicted for *S*^max-neg^_*zz*_ using the B3PW91 functional; as shown when comparing the (b21) and (a21) panels of Fig. 3. From the above presented and discussed evidences, in complete agreement with a variety of experimental observations on the other Ce-based and rare earth based compounds,^27,29,77,78^ we conclude that in the first volume, all the components of the electron-like, as well as the hole-like, Seebeck coefficients of CeRhIn_5_ show a maximum value at low temperatures within the high localized GGA+U and B3PW91 approaches.

The doping levels corresponding to the maximum values of the spin up positive (negative) *xx* and *zz* components of the hole-like (electron-like) Seebeck coefficients, *N*^max-pos^ (*N*^max-neg^), are shown as functions of temperature in Fig. 2(a*i*2) and (b*i*2) (Fig. 3(a*i*2) and (b*i*2)) for *i* = 1–3. In all these hole- and electron-like figures, regardless of the carrier type, the unit of the doping level is evidently electron per unit cell, e per uc, even though the negative carriers are electrons and the positive carriers are holes. We limited the range of the doping level to between −10^21^ and 10^21^ carriers per cm^3^ so that it can be realized in experiments. As can be clearly seen from Fig. 2(a12) and (a22), in the first volume the carriers related to spin up *S*^max-pos^_*xx*_ are electrons, *i.e.*, *N*^max-pos^ < 0, at most of the temperature range within GGA+U and B3PW91 approaches. The same results are observed for the doping level related to spin up *S*^max-neg^_*xx*_ within GGA+U and B3PW91 approaches, see Fig. 3(a12) and (a22). However, the value of *N*^max-pos^ is typically higher than the value of *N*^max-neg^ for the *xx* component in all of the considered temperature range using both GGA+U and B3PW91. For *S*^max-pos^_*zz*_ the related carriers are holes, *i.e.*, *N*^max-pos^ > 0, up to *T* ≃ 45 K within both GGA+U and B3PW91 functionals. However, in the first volume and at *T* ≃ 45 K, a considerable gap suddenly occurs and the *zz*-component of the doping level *N*^max-pos^ drops down from around 1.5 e per uc to about −0.017 e per uc (−1.5 e per uc) within GGA+U (B3PW91), and hence after this temperature the carriers change their type and become electrons, see Fig. 2(b12) (Fig. 2(b22)). After *T* ≃ 45 K, the *zz*-component of the doping level *N*^max-pos^ changes slightly up to room temperature within the B3PW91 functional, see Fig. 2(b22). But, within GGA+U, the *zz*-component of the doping level *N*^max-pos^ again experiences a considerable gap at *T* ≃ 270 K and the doping level drops from around −0.03 e per uc to about −1.6 e per uc. The GGA+U approach predicts that for the electron-like spin up *S*^max-neg^_*zz*_ in the first volume the related carriers are holes up to *T* ≃ 20 K, while at *T* ≃ 20 K a considerable gap occurs and the carriers change their type to electrons up to room temperature, see Fig. 3(a12). In contrast to GGA+U, B3PW91 does not predict a regular behavior for the zz-component of spin up *N*^max-neg^, as can be seen in Fig. 3(b22); there are several considerable gaps in the zz-component of *N*^max-neg^ within the B3PW91. In summary, in most of the temperature range, the predominant carriers related to the *xx*-components of *S*^max-pos^ and *S*^max-neg^, *i.e.*, *N*^max-pos^ and *N*^max-neg^, are electrons using GGA+U and B3PW91, see Fig. 2(a*i*2) and 3(a*i*2) for *i* = 1 and 2. This result holds for the carriers related to the *zz*-components of *S*^max-pos^ within the GGA+U and B3PW91, as shown in the (b*i*2) panels of Fig. 2 for *i* = 1 and 2, as well as for the *zz*-components of *S*^max-neg^ within GGA+U, see Fig. 3(b12). Available experimental reports^24,25,49^ show that applying hydrostatic pressure can improve the Seebeck coefficient of the other Ce-based compounds near room temperature. Furthermore, for strongly correlated f-electron systems the thermoelectric properties, especially the thermopower *S*, are strongly influenced by the position of a very narrow maximum in the density of states relative to the Fermi level. In this case, it is necessary to analyze the calculated *S versus* the change of the Fermi level, due to the high gradient of the density of states at the Fermi level. This analysis can be performed through the imposing pressure, because imposing pressure can change the Fermi level and the DOSs relative to the Fermi level, see Table 2. This motivated us to investigate the effect of pressure on the thermoelectric parameters of the compound under study. Therefore, we have performed our calculations for the two other considered volumes in addition to the first one. For convenience, these volumes are called the second and third volumes from now on. We recall that the second and third volumes are 2% and 5% smaller than the first volume, while the first volume corresponds to the experimental volume. This means that the second and third volumes are under pressure. The results displayed in Fig. 2(a11) and (b11) clearly show that applying pressure within GGA+U does not change *S*^max-pos^_*xx*_ and *S*^max-pos^_*zz*_ drastically for low temperatures, but it makes worse (better) the *S*^max-pos^_*xx*_ (*S*^max-pos^_*zz*_) at high temperatures. Similar results can be obtained for *S*^max-neg^_*xx*_ and *S*^max-neg^_*zz*_. The (a11) panels of Fig. 2 and 3 indicate that within GGA+U the *S*^max-pos^_*xx*_ and *S*^max-neg^_*xx*_ values of the first volume are higher than those of the second and third volumes for *T* > 35 K and *T* > 100 K, respectively. On the contrary, within GGA+U, *S*^max-pos^_*zz*_ and *S*^max-neg^_*zz*_ in the first volume are less than those of the second and third volumes for *T* > 65 K.

As per the result obtained by GGA+U, the volume reduction changes the *S*^max-pos^_*xx*_, *S*^max-neg^_*xx*_ and *S*^max-neg^_*zz*_ values very slightly at low temperatures using B3PW91, however, in contrast to the result obtained by GGA+U, the effect of volume reduction on *S*^max-pos^_*zz*_ at low temperatures is more than high temperatures using B3PW91, see Fig. 2(b21). The maximum values of *S*^max-pos^_*zz*_ in the second and third volumes are about 5.8 and 4.2 μV K^−1^ lower than the maximum value of *S*^max-pos^_*zz*_ in the first volume at *T* ≃ 7 K using B3PW91. For high temperatures, the B3PW91 predicts very different results compared to the GGA+U by applying pressure. In contrast to the GGA+U, the B3PW91 predicts that the *S*^max-pos^_*xx*_ (*S*^max-neg^_*xx*_) in the first volume is less than that of second and third volumes for *T* > 20 K (*T* > 65 K). This effect of pressure is consistent with the experimental report on the Seebeck coefficient of CeIrIn_5_.^24^ Moreover, based on Fig. 3(b21), *S*^max-neg^_*zz*_ is larger in the first volume than that in the second volume but lower than that in the third volume for *T* > 65 K. The volume reduction effect on *S*^max-pos^_*zz*_ is negligible at high temperatures. The effect of volume reduction on both components of *N*^max-pos^ (*N*^max-neg^) doping levels is significant only at low temperatures using GGA+U, see the (a12) and (b12) panels of Fig. 2 (Fig. 3). The *xx* and *zz* components of *N*^max-pos^ (*N*^max-neg^) remain approximately unchanged by the volume reduction for *T* > 35 K (*T* > 20 K) and *T* > 45 K (*T* > 36 K), respectively. On the other hand, B3PW91 predicts that both components of *N*^max-pos^ and *N*^max-neg^ doping levels in the first volume are significantly different from the second and third volumes in most of the considered temperature range, see the (a22) and (b22) panels of Fig. 2 and 3. But, the doping levels in the second and third volumes are very similar for both components and both electron- and hole-like Seebeck coefficient using B3PW91.

Shishido and coworkers,^50,51^ using dHvA experiments, have shown that the degree of 4f-electron localization has been decreased by imposing pressure on CeRhIn_5_. Hence, it is interesting to investigate the localization effects on the thermoelectric parameters of CeRhIn_5_. Moreover, variation of the Hubbard U parameter can strongly affect the calculated thermoelectric properties. Thus, we have calculated these parameters using three different functionals with three different degrees of localization, *i.e.*, PBE-GGA, GGA+U and B3PW91, to study the effects of the degree of localization on the thermoelectric parameters. The degree of 4f-Ce electron localization is predicted to be much lower by PBE-GGA than GGA+U and B3PW91 schemes. The *xx* and *zz* components of *S*^max-pos^ (*S*^max-neg^), as calculated by PBE-GGA, are shown in the (a31) and (b31) panels of Fig. 2 (Fig. 3) for the three considered volumes. PBE-GGA predicts a peak with a value of about 6.7 μV K^−1^ (4.9 μV K^−1^) for *S*^max-pos^_*xx*_ (*S*^max-pos^_*zz*_) at *T* ≃ 7 K (*T* ≃ 10 K) in the first volume, as shown in Fig. 2(a31) (Fig. 2(b31)). This prediction of the low localized PBE-GGA is very close to the predictions of the high localized GGA+U and B3PW91. After this temperature (T ≃ 7 K), where the maximums occur in the Seebeck curves, the *S*^max-pos^_*xx*_ (*S*^max-pos^_*zz*_) decreases to a value of about 5.4 μV K^−1^ (4.3 μV K^−1^) at around *T* ≃ 20 K, and then it increases again as temperature increases up to the room temperature. These results indicate that the behavior of *S*^max-pos^_*xx*_ and *S*^max-pos^_*zz*_ predicted by the low localized PBE-GGA is the same as those predicted by the high localized GGA+U and B3PW91 at low temperatures. Consequently, it can be concluded that at low temperatures the degree of 4f-Ce localization has no considerable effect on the *S*^max-pos^_*xx*_ and *S*^max-pos^_*zz*_ values. But, the low localized PBE-GGA predicts much higher values for the *S*^max-pos^_*xx*_ and *S*^max-pos^_*zz*_ values at high temperatures compared to the high localized GGA+U and B3PW91. This can be clearly seen for the *xx* [*zz*] component by comparing the (a31) [(b31)] panel with the (a11) [(b11)] and (a21) [(b21)] panels of Fig. 2. This comparison also indicates that within the low localized PBE-GGA, the values of *S*^max-pos^_*xx*_ at high temperatures are about four times larger than those at low temperatures, while its values are in the same range at low and at high temperatures using the high localized GGA+U and B3PW91. This result also holds for *S*^max-pos^_*zz*_. Therefore, the maximum values of *S*^max-pos^ components as predicted by PBE-GGA at low temperatures can be approximately neglected compared to the 4 times higher values of *S*^max-pos^ components at high temperatures. In this case, we can assume that the PBE-GGA Seebeck curves monotonically increase by temperature.

The monotonic increase of the Seebeck curves by temperature is a well-known character of normal metals. Therefore, CeRhIn_5_ is estimated by PBE-GGA to behave almost like a normal metal, if the small peaks at low temperatures are omitted in the (a31) and (b31) panels of Fig. 2 compared to the large values of the Seebeck components at high temperatures. The effect of volume reduction also depends on the functional used so that it is differently predicted by PBE-GGA compared to GGA+U and B3PW91. Fig. 2(a31) and (b31) reveal that volume reduction makes worse both the *xx* and *zz* components of *S*^max-pos^ at high temperatures. The same result can be also seen for the *xx* component at low temperatures, because the PBE-GGA *xx* component of *S*^max-pos^ in the first volume is more than those in the second and third volumes, see Fig. 2(a31). Thus, volume reduction decreases the *xx* component of *S*^max-pos^ and as a result makes it worse. But, the *zz* component is slightly improved by volume reduction at low temperatures using PBE-GGA. In contrast to *S*^max-pos^, the *S*^max-neg^ components are completely zero at high temperatures using PBE-GGA, see Fig. 3(a31) and (b31). This zero value implies that it may be impossible to find the electron-like Seebeck coefficient using PBE-GGA at the considered doping level range. Fig. 3(a31) and (b31) show that PBE-GGA, the same as GGA+U and B3PW91, predicts a maximum (negative minimum) for the *xx* and *zz* components of *S*^max-neg^ at low temperatures, *i.e.*, *T* ≃ 7 K in the first volume. The latter figures also show that the volume reduction slightly improves the *xx* and *zz* components of *S*^max-neg^ at low temperatures within PBE-GGA, but does not change their zero values at high temperatures. In summary, a comparison of the (a*i*1) [(b*i*1)] panels of Fig. 3 for *i* = 1–3 demonstrates that reduction of the localization degree considerably influences *S*^max-neg^_*xx*_ (*S*^max-neg^_*zz*_) at high temperatures, but it is not very remarkable at low temperatures. The doping levels related to the *xx* and *zz* components of *S*^max-pos^ (*S*^max-neg^) predicted using PBE-GGA, are displayed in the (a32) and (b32) panels of Fig. 2 (Fig. 3). Both components of *N*^max-pos^ are approximately fixed at most of the temperature range, except for a considerable gap at low temperatures, as shown in Fig. 2(a32). The latter figures also show that the volume reduction does not considerably change the doping levels. For most of the temperature range, PBE-GGA predicts about 1.5 e per uc electron doping for the *S*^max-pos^ components in the three considered volumes. The *xx* [*zz*] component of *N*^max-pos^ is not considerably affected by decreasing the localization degree, see the (a32) [(b32)] panel and the (a12) [(b12)] and (a22) [(b22)] panels of Fig. 2.

We also see considerable gaps in the *N*^max-neg^ components at low temperatures, within PBE-GGA in three volumes, see Fig. 3(a32) and (b23). We do not show any doping level, *N*^max-neg^, in the latter figures for temperatures in which the *S*^max-neg^ components are zero, because the doping level for this situation is meaningless. At the end of this section and based on the presented results, we can conclude that for the first volume corresponding to the experimental volume, both *xx* and *zz* Seebeck coefficient components of CeRhIn_5_ show maximum values at low temperatures around the *T* ≃ 7 K using the three considered XCFs. Based on our results, the volume reduction does not affect the Seebeck coefficient components considerably at low temperatures, except for *S*^max-pos^_*zz*_. But, this is not the case for high temperatures. The effect of volume reduction depends on the functionals used, in agreement with our recent report.^55^ The volume reduction makes worse the Seebeck coefficient component along the *a* crystalline axis within the GGA+U and PBE-GGA approaches at high temperatures. Along the *c* crystalline axis, volume reduction improves the Seebeck coefficient component at high temperatures within GGA+U, but makes it worse within PBE-GGA. On the contrary, the *xx* components of the hole- and electron-like Seebeck coefficients, as calculated by B3PW91, along the *a* (*c*) crystalline axis in the first volume are less (more) than those in the second and third volumes at high (low) temperatures. Our results also show that the degree of localization for the 4f-Ce electrons has a significant effect on the Seebeck coefficient components at high temperatures, but at low temperatures these effects are negligible. Decreasing the Seebeck coefficient causes the hole like Seebeck coefficient components of heavy fermion CeRhIn_5_ to almost behave like the normal metals. Moreover, the doping levels related to the maximum values of the hole-like and electron-like components are electrons at most of the considered temperature range in all the considered volumes and XCFs.

## 5 Electrical conductivity

**Fig. 4:**
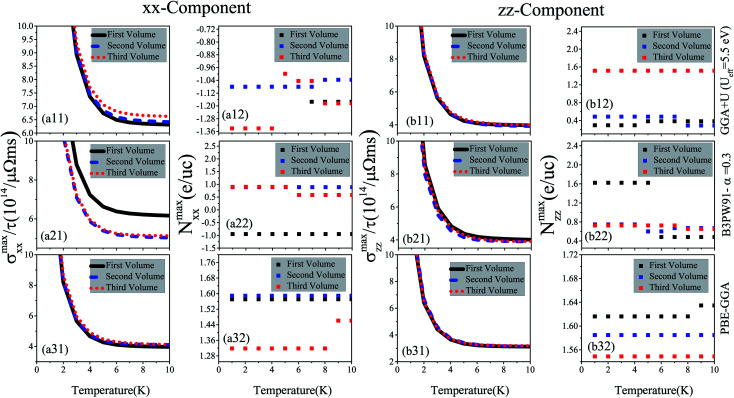
Maximum values of (a*i*1) *xx* and (b*i*1) *zz* components of spin up electrical conductivity per relaxation time (*τ*), *i.e.*, (a*i*1) *σ*^max^_*xx*_/*τ* and (b*i*1) *σ*^max^_*zz*_/*τ*, *versus* temperature calculated using GGA+U with *U*_eff_ = 5.5 eV, B3PW91 with *α* = 0.30 and PBE-GGA functionals in the three different volumes at low temperatures (*T* ≤ 10 K). The first volume corresponds to the experimental volume at zero pressure. The second and third volumes are 2% and 5% smaller than the first volume, respectively. Doping levels related to *σ*^max^_*xx*_/*τ* (*N*^max^_*xx*_ (e per uc)) and *σ*^max^_*zz*_/*τ* (*N*^max^_*zz*_ (e per uc)) are displayed in the (a*i*2) and (b*i*2) panels, respectively. The *i*-index varies from 1 to 3.

**Fig. 5:**
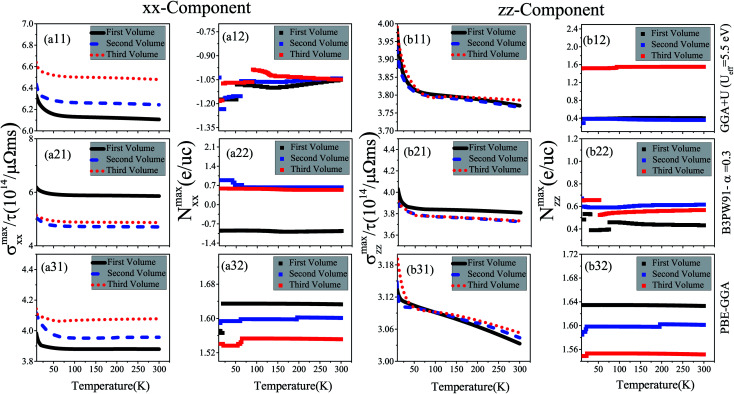
Maximum values of (a*i*1) *xx* and (b*i*1) *zz* components of spin up electrical conductivity per relaxation time (*τ*), *i.e.*, (a*i*1) *σ*^max^_*xx*_/*τ* and (b*i*1) *σ*^max^_*zz*_/*τ*, *versus* temperature calculated using GGA+U with *U*_eff_ = 5.5 eV, B3PW91 with *α* = 0.30 and PBE-GGA functionals in the three different volumes at high temperature (10 K ≤ *T* ≤ 300 K). The first volume corresponds to the experimental volume at zero pressure. The second and third volumes are 2% and 5% smaller than the first volume, respectively. Doping levels related to *σ*^max^_*xx*_/*τ* (*N*^max^_*xx*_ (e per uc)) and *σ*^max^_*zz*_/*τ* (*N*^max^_*zz*_ (e per uc)) are displayed in the (a*i*2) and (b*i*2) panels, respectively. The *i*-index varies from 1 to 3.

Electrical conductivity is another important quantity that affects the thermoelectric efficiency. According to the definition of the figure of merit, higher values of electrical conductivity can improve thermoelectric efficiency. As expressed in eqn (1), the electrical conductivity, *σ*(*μ*,*T*), is a tensor of rank 2 with nine components. However, similar to the Seebeck coefficient, we only investigate the *xx* and *zz* components of *σ*(*μ*,*T*). In the Boltzmann equations, the electrical conductivity is a function of relaxation time (*τ*), see eqn (1). Thus, in this study we discuss the electrical conductivity per relaxation time, *i.e.*, *σ*(*μ*,*T*)/*τ*. The maximum values of the *xx* and *zz* components of *σ*(*μ*,*T*)/*τ*, *i.e.*, *σ*^max^_*xx*_/*τ* and *σ*^max^_*zz*_/*τ* calculated using the three GGA+U, B3PW91 and PBE-GGA XCFs for CeRhIn_5_ are shown in the (a*i*1) and (b*i*1) panels of Fig. 4 in the three considered volumes *versus* temperature at low temperatures, *i.e.*, *T* ≤ 10 K. The results of the high temperatures, *i.e.*, 10 K ≤ *T* ≤ 300 K, are displayed in Fig. 5. The results show that both *xx* and *zz* components of *σ*^max^/*τ* decrease as temperature increases at low temperatures for the three considered volumes, see Fig. 4(a*i*1) and (b*i*1) for *i* = 1–3. The components of *σ*^max^/*τ* also decrease by increase of temperature at high temperatures, but the slopes of *σ*^max^/*τ* components at high temperatures are much lower than those at low temperatures, see Fig. 5(x*i*1) and Fig. 4(x*i*1) for x = a & b and *i* = 1–3. These results are in agreement with the experimental results.^43^ As can be clearly seen from Fig. 4(a11), the *σ*^max^_*xx*_/*τ* value slightly increases by volume reduction for *T* < 10 K using GGA+U. In contrast to GGA+U, *σ*^max^_*xx*_/*τ* considerably decreases by the volume reduction from the first to the second or third volume for the *T* < 10 K within the B3PW91 XCF, see Fig. 4(b21). But, *σ*^max^_*xx*_/*τ* in the second and third volumes are very similar within B3PW91. Within the PBE-GGA XCF the *xx* component does not change drastically by the volume reduction at low temperatures, see Fig. 4(a31). The same results can be reached at high temperatures, see Fig. 5(a*i*1). As an important result, comparing the results of GGA+U, B3PW91 and PBE-GGA reveals that the effect of volume reduction on *σ*^max^_*xx*_/*τ* strongly depends on the degree of localization of 4f-Ce electrons, consistent with our previous work.^55^ But this is not the case for *σ*^max^_*zz*_/*τ*, *viz.* the *σ*^max^_*zz*_/*τ* is not drastically changed by the volume reduction using all the considered XCFs, see Fig. 4(b*i*1) [Fig. 5(b*i*1)] *i* = 1–3 for low [high] temperatures. Moreover, *σ*^max^_*xx*_/*τ* [*σ*^max^_*zz*_/*τ*] is predicted to be slightly larger by the high localized GGA+U and B3PW91 than by the low localized PBE-GGA at low temperatures, see Fig. 4(a*i*1) [Fig. 4(b*i*1)] (*i* = 1–3). Similar results can be observed for the *xx* [*zz*] component at high temperatures through the comparison of Fig. 5(a*i*1) [Fig. 5(b*i*1)] (*i* = 1–3).

The doping levels related to the components of the maximum electrical conductivity are presented in Fig. 4(a*i*2) and (b*i*2) for *i* = 1–3. As shown in Fig. 4(a12) and 5(a12), the doping carriers related to *σ*^max^_*xx*_/*τ* are electrons for all the temperature range in the three considered volumes using GGA+U. B3PW91 predicts that the doping levels corresponding to the *σ*^max^_*xx*_/*τ* are electron (hole) for all the temperature range in the first (second and third) volume(s), see Fig. 4(a22) and 5(a22). PBE-GGA predicts the hole doping levels corresponding to the *σ*^max^_*xx*_/*τ* for all the temperature range for the three considered volumes, as shown in Fig. 4(a32) and 5(a32). These results confirm that the volume reduction effects on the doping levels related to *σ*^max^_*xx*_/*τ* depend on the degree of localization for 4f-Ce electrons. For *σ*^max^_*zz*_/*τ*, the doping levels are holes using all the considered XCFs at all the temperature range, see (b*i*2) panels of Fig. 4 and 5 for *i* = 1–3.

## 6 Thermal conductivity

**Fig. 6:**
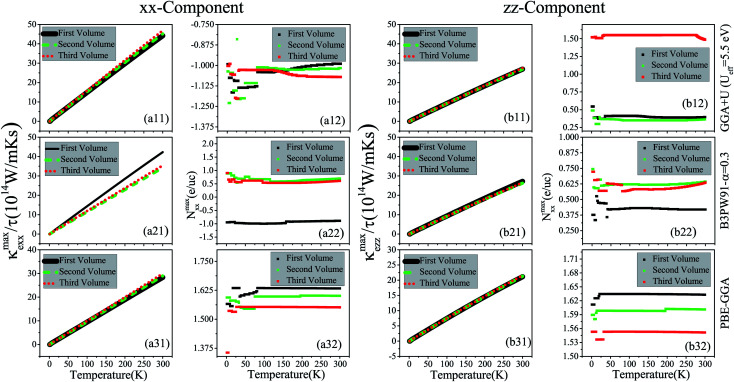
Maximum values of (a*i*1) *xx* and (b*i*1) *zz* components of spin up electronic thermal conductivity per relaxation time (*τ*), *i.e.*, (a*i*1) *κ*^max^_e*xx*_/*τ* and (b*i*1) *κ*^max^_e*zz*_/*τ versus* temperature calculated using GGA+U with *U*_eff_ = 5.5 eV, B3PW91 with *α* = 0.30 and PBE-GGA functionals in the three different volumes. The first volume corresponds to the experimental volume at zero pressure. The second and third volumes are 2% and 5% smaller than the first volume, respectively. Doping levels related to *κ*^max^_e*xx*_/*τ* and *κ*^max^_e*zz*_/*τ* and are displayed in the (a*i*2) and (b*i*2) panels, respectively. The *i*-index varies from 1 to 3.

Thermal conductivity is another parameter that can affect the thermoelectricity. Thermal conductivity includes lattice and electronic contributions. Here, we only concentrate on the electronic part, because in metals the lattice part constitutes only a small fraction of the total thermal conductivity (less than 2 percent).^79,80^ Therefore, we use the character of *Z*_e_*T* as the electronic figure of merit to investigate the thermoelectric efficiency of our case along several directions, where 
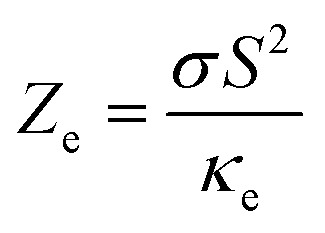
. Similar to the electrical conductivity, thermal conductivity is a tensor of rank two which depends on the relaxation time, as expressed in eqn (3). The maximum values of the electronic thermal conductivity per relaxation time, *κ*^max^_e_/*τ* along the *a* and *c* crystalline axes calculated using GGA+U, B3PW91 and PBE-GGA XCFs are plotted *versus* temperature in the (a*i*1) and (b*i*1) panels of Fig. 6 for the first, second and third volumes. The results show that the maximum values of the electronic thermal conductivity components increase as temperature increases for all the considered volumes, see Fig. 6(a*i*1) and (b*i*1) for *i* = 1–3. By comparing Fig. 6(a*i*1) and (b*i*1), it can be observed that in the three considered volumes the *xx* and *zz* components of *κ*^max^_e_/*τ* are of the same order of magnitude. According to our results, the volume reduction does not drastically change the *xx* and *zz* components of *κ*^max^_e_/*τ*, apart from the *xx* component calculated by B3PW91 at high temperatures.

The values of doping levels corresponding to the maximum values of *κ*^max^_e_/*τ* components are presented in the (a*i*2) and (b*i*2) panels of Fig. 6 for *i* = 1–3. As shown in Fig. 6(a12), the electron doping levels can be attributed to the *xx* component of *κ*^max^_e_/*τ* for all the temperature range in the three considered volumes using the GGA+U approach. The same result can be observed using B3PW91 for the *xx* component of *κ*^max^_e_/*τ* in all the temperature range in the first volume, see Fig. 6(a22). However, this is completely different for the *xx* component of *κ*^max^_e_/*τ* in the second and third volumes using B3PW91 XCF, as shown in Fig. 6(a22). As the latter figure shows, in all the considered temperature range, the hole doping is related to the *xx* component of *κ*^max^_e_/*τ* in the second and third volumes using B3PW91. The hole doping levels are related to the *xx* component of *κ*^max^_e_/*τ* in the three considered volumes using PBE-GGA in all the considered temperature range, as shown in Fig. 6(a32). Similar results can be observed for the electrical conductivity. All the considered XCFs predict the hole doping levels for the *zz* component of *κ*^max^_e_/*τ* in the three considered volumes, as shown in the (b*i*2) panels of Fig. 6 for *i* = 1–3.

## 7 Power factor

**Fig. 7:**
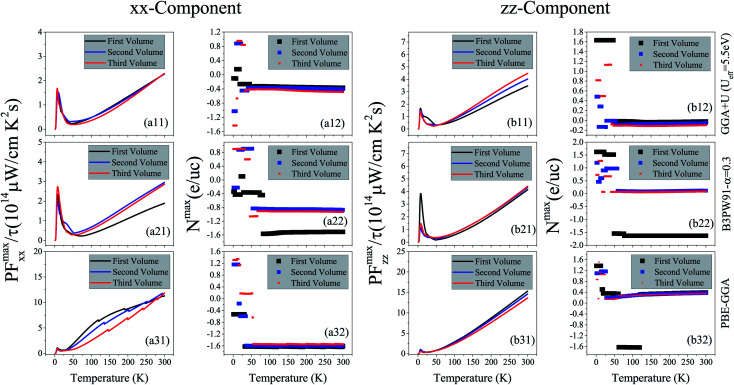
Maximum values of (a*i*1) *xx* and (b*i*1) *zz* components of spin up power factor per relaxation time (*τ*), *i.e.*, (a*i*1) PF^max^_*xx*_/*τ* and (b*i*1) PF^max^_*zz*_/*τ versus* temperature calculated using GGA+U with *U*_eff_ = 5.5 eV, B3PW91 with *α* = 0.30 and PBE-GGA functionals in the three different volumes. The first volume corresponds to the experimental volume at zero pressure. The second and third volumes are 2% and 5% smaller than the first volume, respectively. Doping levels related to PF^max-pos^_*xx*_/*τ* and PF^max-pos^_*zz*_/*τ* are displayed in the (a*i*2) and (b*i*2) panels, respectively. The *i*-index varies from 1 to 3.

**Fig. 8:**
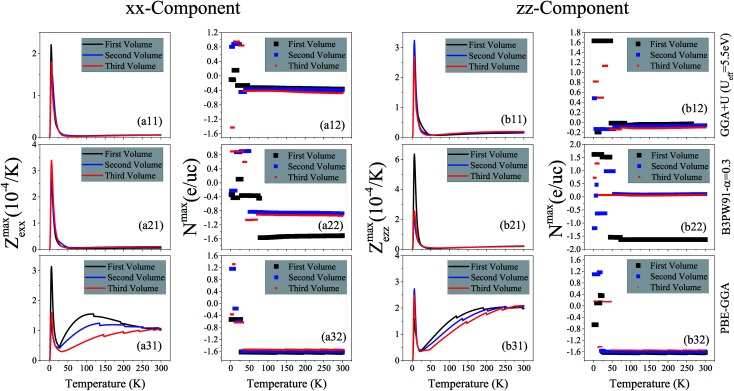
Maximum values of (a*i*1) *xx* and (b*i*1) *zz* components of electronic figure of merit per temperature, *i.e.*, (a*i*1) *Z*^max^_e*xx*_ and (b*i*1) *Z*^max^_e*zz*_*versus* temperature calculated using GGA+U with *U*_eff_ = 5.5 eV, B3PW91 with *α* = 0.30 and PBE-GGA functionals in the three different volumes. The first volume corresponds to the experimental volume at zero pressure. The second and third volumes are 2% and 5% smaller than the first volume, respectively. Doping levels related to *Z*^max^_e*xx*_ and *Z*^max^_e*zz*_ are displayed in the (a*i*2) and (b*i*2) panels, respectively. The *i*-index varies from 1 to 3.

The numerator of *ZT*, *i.e.*, *σS*^2^, is known as the power factor (PF). Since the electrical conductivity in the Boltzmann equations is calculated by means of the relaxation time approximation, the PF also depends on the relaxation time and thereby we consider PF/*τ*. The maximum values of the *xx* and *zz* components of the power factor of the compound per *τ*, PF^max^/*τ*, calculated using GGA+U, B3PW91 and PBE-GGA XCFs in the first, second and third volumes, are shown as functions of temperature in Fig. 7(a*i*1) and (b*i*1) for *i* = 1–3. As can be clearly seen in Fig. 7(a*i*1) and (b*i*1) for *i* = 1–2, at the experimental zero pressure, *i.e.*, (the first volume), there are considerable peaks for the *xx* and *zz* components of PF^max^, *i.e.*, PF^max^_*xx*_ and PF^max^_*zz*_, at low temperatures around *T* = 7 K using the GGA+U and B3PW91 approaches. After *T* = 7 K, the GGA+U (B3PW91) predicts that the PF^max^_*xx*_ and PF^max^_*zz*_ values decrease as temperature increases up to about *T* = 45 K (*T* = 70 K), and then they increase as temperature increases up to about room temperature. One also would notice that in the first volume, the *zz* component of PF^max^ is slightly more than the *xx* component using both high localized GGA+U and B3PW91 methods, specifically at high temperatures. As Fig. 7(a11) and (b11) display, the GGA+U predicts that volume reduction does not change PF^max^_*xx*_ considerably, but PF^max^_*zz*_ slightly increases (decreases) at high (low) temperatures by the volume reduction. The B3PW91 XCF predicts that at low temperatures, PF^max^_*zz*_ in the first volume is the same as the second and third volumes, but at high temperatures (*T* > 50 K), PF^max^_*xx*_ in the first volume is considerably less than the volumes under pressure, *i.e.*, the second and third volumes, see Fig. 7(a21). The volume reduction effect on PF^max^_*zz*_ is completely different within B3PW91; at low temperatures, PF^max^_*zz*_ in the first volume is considerably more than the second and third volumes, but at high temperatures PF^max^_*zz*_ in the first volume is the same as the second and third volumes, see Fig. 7(b21). The same as the high localized GGA+U and B3PW91 approaches, the low localized PBE-GGA predicts a peak for PF^max^_*xx*_ and another peak for PF^max^_*zz*_ at low temperature (*T* ≃ 7 K) for the three considered volumes. But, the values of the peaks are negligible compared to the high values of PF^max^ components at high temperatures. By ignoring the negligible peak at low temperatures, the behavior of the PF^max^ components of CeRhIn_5_ using PBE-GGA is very similar to the behavior of the PF^max^ components of a normal metal, *i.e.*, PF^max^ components increase as temperature increases. As shown in Fig. 7(a31), the PF^max^_*xx*_ value calculated by PBE-GGA decreases as volume decreases, specifically at high temperatures. The same results can be seen for PF^max^_*zz*_, see Fig. 7(b31), however, the effect of volume reduction on the PF^max^_*xx*_ value is more considerable compared to that of PF^max^_*zz*_. The comparison of Fig. 7(a*i*1) for *i* = 1–3 shows that PF^max^_*xx*_ considerably increases as the degree of localization decreases in all the considered volumes at high temperatures but not significantly at low temperatures. Similar result can be seen for PF^max^_*zz*_ by the comparison of Fig. 7(bil) for *i* = 1–3. The doping levels corresponding to PF^max^ components in the three considered volumes are shown in the (a*i*2) and (b*i*2) panels of Fig. 7 (*i* = 1–3) for the *xx* and *zz* components, respectively. Based on Fig. 7(a21), the low level of electron doping (about 0.3 e per uc) originates from PF^max^_*xx*_ in most of the temperature range (in the first volume) using GGA+U. The same result can be seen for PF^max^_*zz*_ at zero pressure and *T* ≥ 50 K. The types of the doping level related to PF^max^_*zz*_ are electrons with about 0.03 e per uc concentration at most of the considered temperature range (*T* ≥ 50 K) in the first volume using GGA+U. But, at low temperatures, the doping levels related to PF^max^_*zz*_ are holes with about 1.6 e per uc in the first volume using GGA+U. As with the first volume, we see very low doping concentration of electrons for both *xx* and *zz* components of PF^max^ at the considered nonzero pressures, *i.e.*, in the second and third volumes at most of the considered temperature range within the GGA+U. B3PW91 predicts the electron doping levels for PF^max^_*xx*_ in most of the temperature range in the first volume, as shown in Fig. 7(a22). This Figure also indicates that imposing pressure within B3PW91 decreases the values of doping levels related to PF^max^_*xx*_ and PF^max^_*zz*_ at most of the temperature range. The same result is reached for PF^max^_*zz*_, see Fig. 7(b22). Moreover, imposing pressure using B3PW91 changes the type of the doping levels related to PF^max^_*zz*_, while this is not the case for PF^max^_*xx*_. In summary, we see a very low concentration of electron (hole) doping related to PF^max^_*xx*_ (PF^max^_*zz*_) at most of the temperature range in the second and third volumes using B3PW91. The same as the high localized GGA+U and B3PW91 XCFs, the low localized PBE-GGA predicts the electron doping level corresponding to the PF^max^_*xx*_ at most of the temperature range, as shown in Fig. 7(a32). On the contrary, PBE-GGA predicts a hole doping level corresponding to the *zz* component of PF^max^ at most of the temperature range, see Fig. 7(b32). Fig. 7(a32) and (b32) show that the volume reduction does not change drastically the *xx* and *zz* components of the doping level using PBE-GGA the same as GGA+U. In summary, we see that the behavior of the *xx* and *zz* components of PF^max^ is very similar to those of the *S*^max-pos^, see Fig. 7(a*i*1) and (b*i*1) and Fig. 2(a*i*1) and (b*i*1). Within the high localized XCFs, the *xx* and *zz* components of PF^max^ show a peak at low temperatures. Furthermore, the values of PF^max^ components at low and at high temperatures are in the same range. On the contrary within the low localized PBE-GGA, the values of PF^max^ components at high temperatures are much higher than those at low temperatures. Our results show that in the experimental zero pressure (the first volume) the doping levels related to both components of PF^max^ are electrons within all the considered XCFs, except for the *zz* component using PBE-GGA. Furthermore, our results show that the volume reduction does not change the type and values of the doping levels within GGA+U and PBE-GGA, while this is not the case within the B3PW91 XCF. Within the B3PW91 XCF, the doping level is decreased by the volume reduction for most of the temperature range. Moreover, imposing pressure within B3PW91 changes the type of the doping levels related to PF^max^_*zz*_. In fact, the efficiency of thermoelectricity depends on both PF and thermal conductivity, *viz.*
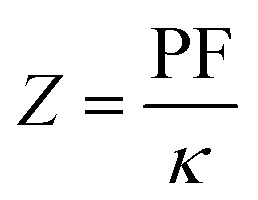
. As indicated before, here, we only calculate the electronic part of the thermal conductivity by introducing *Z*_e_*T* as electronic figure of merit to investigate the thermoelectric efficiency of our case, where 
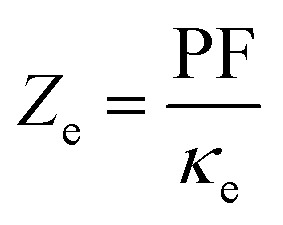
. In general, *Z*_e_ differs from *Z*, because the lattice part of thermal conductivity is ignored in *Z*_e_. However, in metals the lattice part is very small and can be safely neglected.^79,80^ The maximum values of the *xx* and *zz* components of *Z*^max^_e_ are shown in the (a*i*1) and (b*i*1) panels of Fig. 8 for *i* = 1–3. The (a*i*2) and (b*i*2) panels of this figure show the doping levels corresponding to the *xx* and *zz* components of *Z*^max^_e_. As shown in the (a11) and (b11) panels of Fig. 8, the GGA+U approach predicts considerable peaks for both *xx* and *zz* components of *Z*^max^_e_ at low temperatures in the three considered volumes. Moreover, the height of this peak for the *zz* component is slightly higher than that of the *xx* component. The same results can be reached within the B3PW91 XCF, see the (a21) and (b21) panels of Fig. 8. These peaks are also predicted using the low localized PBE-GGA, see Fig. 8(a31) and (b31). The effect of imposing pressure on these peaks strongly depends on the used XCF. The B3PW91 XCF predicts that the height of the *xx* (*zz*) component peak is increased (decreased) by imposing pressure, but in contrast GGA+U and PBE-GGA predict that the *xx* (*zz*) component peak decreases (increases) as pressure increases. Comparing Fig. 8(a31) with the Fig. 8(a11) and (a21) reveals that decreasing the degree of localization considerably increases the *xx* component of *Z*^max^_e_ at high temperature. The same result is seen for the *zz* component by comparison of Fig. 8(b31) with Fig. 8(b11) and (b21). The (a*i*2) and (b*i*2) panels of the latter figure show that the doping levels related to *Z*^max^_e_ are electrons at most of the temperature range within all the used XCFs in the three considered volumes.

## 8 Conclusions

Thermoelectric properties and performance as well as electronic structures of the heavy fermion CeRhIn_5_ are studied at different pressures employing density functional and semiclassical Boltzmann theories utilizing our developed physical and practical scheme applied to the well-known BoltzTraP code for describing the behaviors of the quantities as functions of temperature. It is found that the 4f-Ce electrons play an important role in the properties of this compound. It is shown that the electronic structure of the system can be also affected by pressure. The thermoelectric results reveal that the maximum values of the hole-like (positive) and electron-like (negative) Seebeck coefficients occur at low temperatures by the highly localized exchange–correlation functionals (XCFs) used here. This prediction is consistent with the available experimental Seebeck coefficients of the other Ce-based compounds. In contrast, the maximum values of both Seebeck coefficients of the case increase as temperature increases using band-like PBE-GGA, and thereby the global maximum values occur at room temperature as the highest temperature considered in this study for the hole-like Seebeck coefficient. In fact, PBE-GGA predicts a normal metal behavior for the maximum values of the hole-like Seebeck coefficient components. Thus, our results reveal that decreasing the degree of 4f-Ce localization makes the Seebeck coefficient better near room temperature, but does not change it significantly at lower temperatures. We also find the behavior of power factor (PF) components very similar to that of the hole-like Seebeck coefficient components so that decreasing the 4f-Ce degree of localization can make the maximum values of PF components better near room temperature, but does not change them significantly at lower temperatures. Electrical conductivity (electronic part of thermal conductivity) calculations reveal that the maximum of this quantity decreases (increases) as temperature increases at all the considered pressures within all of the considered XCFs. These calculations also reveal the maximum values of the *σ* component (*κ*_e_ component) along the *a* crystalline axis are slightly higher than those along the *c* crystalline axis. Furthermore, the maximum value of *σ* and *κ*_e_ components are slightly decreased by decreasing the degree of localization for 4f-Ce electrons. The type of doping levels related to the *σ* component and *κ*_e_ component along the *a* crystalline axis at the zero and non-zero pressures is electron-like for most of the temperature range using the high localized GGA+U, while this is hole-like using the low localized PBE-GGA. The type of doping levels related to the *σ* component and *κ*_e_ component along the *a* crystalline axis is electron-like for most of the temperature range at zero pressure using B3PW91, but the type is changed by imposing pressure to the hole-like. For the components of *σ* and *κ*_e_ along the *c* crystalline axis the related doping levels are holes for most of the temperature range for zero as well as non-zero pressures regardless of the used XCF. We also investigate the thermoelectric efficiency (*Z*_e_) quantity. Our results show that the *Z*_e_ of our considered case along the *a* crystalline axis is slightly less than that of the *c* axis specifically at low temperatures. This study shows that the thermoelectric efficiency of CeRhIn_5_ decreases as temperature increases and the efficiency can be improved by decreasing the degree of localization for the 4f-Ce electrons near the room temperature.

## Conflicts of interest

There are no conflicts to declare.

## Supplementary Material

RA-009-C9RA07859B-s001
