# Supplementary material for: Thermoelectric properties of heavy fermion CeRhIn5 using density functional theory combined with semiclassical Boltzmann theory
Source: RSC Adv. 2019 Nov 6;9(62):36182–97. doi: 10.1039/c9ra07859b (PMC9074952; doi:10.1039/c9ra07859b)
Supplement: RA-009-C9RA07859B-s001 [file RA-009-C9RA07859B-s001.pdf]

Electronic Supplementary Material (ESI) for RSC Advances.
This journal is © The Royal Society of Chemistry 2019

RSC Advances

Electronic Supplementary Information (ESI)

Cite this: DOI: 10.1039/xxxxxxxxxx

Electronic supplementary information (ESI) for the article entitled "Thermoelectric properties of heavy fermion $CeRhIn_5$ using density functional theory combined with semiclassical Boltzmann theory"

M. Yazdani-Kachoei,[a] and S. Jalali-Asadabadi*[a]

Received Date
Accepted Date

DOI: 10.1039/xxxxxxxxxx

www.rsc.org/journalname

This electronic supplementary information (ESI) is provided to support the results and discussion presented in the main paper. The calculated band structures and densities of states (DOSs) are presented using three different exchange-correlation functionals with three different degrees of localization. Then, the Kohen-Sham Hamiltonian using each of these functionals is applied to study the electronic structures of $CeRhIn_5$ with three different volumes. Thus, 9 band structures and 9 DOSs are shown for spin up. Similarly, 9 band structures and 9 DOSs are presented for spin down. These 36 figures are presented in this ESI to concise and make straightforward the electronic structure discussion of the main paper. Instead in the main paper, extracted quantitative values from these figures are quantitatively discussed to increase the accuracy of the discussion. In this ESI, two computer programs and a flowchart are also appended to numerically support thermoelectric values presented in the main paper. This will show how the data are extracted from the output file of BoltzTrap code and make them easily reproducible by the others. These programs are provided because in most of the regular thermoelectric studies by BoltzTrap using its default outputs, the thermoelectric properties are not presented versus temperature. However, our calculated thermoelectric properties are plotted here as functions of temperature due to the main goal of the paper which is determining the thermoelectric efficiency of the system under study.

1 Crystal structure

The chemical structure of the $CeRhIn_5$ compound, as shown in Fig. 1, crystallizes in P4/mmm space group number 123.[1–3] . The experimental lattice parameters of the compound were measured to be $a = 4.656$ (Å) and $c = 7.542$ (Å).[2,3]

2 Post processing calculations

$CeRhIn_5$ is a well-known heavy-fermion antiferromagnet at room temperature which crystallizes in P4/mmm space group number 123.[1–3] The chemical structure of this material is shown in Fig. 1.

3 Electronic structure

We have calculated the band structures using PBE-GGA, PBE-GGA+U with U_{eff}=5.5 eV, and hybrid B3PW91 with α=0.3 for $CeRhIn_5$ compound having three different volumes. The first volume is the experimental volume at zero pressure. The second and third volumes are 2% and 5% smaller than the first volume, respectively. The calculated band structures are presented in Figs. 2 and 3, respectively. In these figures, the E_F is set to zero. Furthermore, contributions of the 4f-Ce states are characterized by the thickness of the bands; the larger the band thickness, the higher the contribution of the 4f-Ce states to the band.

The results show that the pressure cannot considerably change the band structures of the system, as can be seen from any rows of Fig. 2 or Fig. 3. For example, if we consider the first row of Fig. 2, we clearly observe that most of the 4f-Ce states are very closely distributed around the Fermi level in Figs. 2(a11), (a12) and (a13). This can be also seen in the second and third rows of Fig. 2 or Fig. 3. This observation implies that the distribution of the 4f-Ce states is not significantly changed when we apply pressure theoretically withoth considering the changes made in the degree of localization due to our applied pressure. In contrast to the pressure, however, the degree of localization can more considerably affect the band structures, as can be seen from any columns of Fig. 2 or Fig. 3. For example, if we consider the first column of Fig. 2, we clearly observe that the distribution of the

[a] Department of Physics, Faculty of Sciences, University of Isfahan (UI), Hezar Gerib Avenue, Isfahan 81746-73441, Iran. Fax: 98 31 37934800; Tel: 98 31 37932435; E-mail: saeid.jalali.asadabadi@gmail.com; sjalali@sci.ui.ac.ir

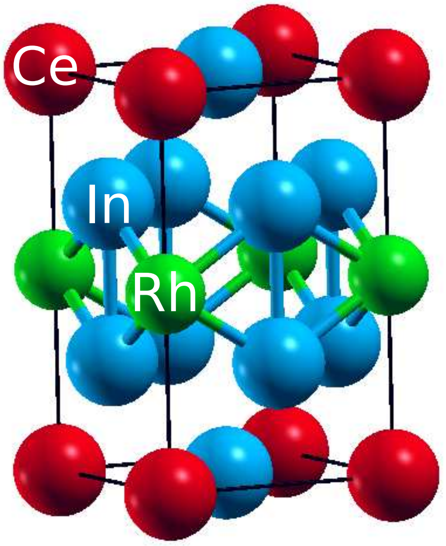

Fig. 1 Chemical structure of the $CeRhIn_5$ compound.

4f-Ce states is considerably affected by changing the functionals and thereby the degree of localization in Figs. 2(a11), (a21) and (a31). This can be also seen in the second and third columns of Fig. 2 or Fig. 3.

Total and 4f-Ce as well as 4d-Rh partials DOSs of $CeRhIn_5$ are also calculated using PBE-GGA, GGA+U with U_{eff}=5.5 eV, and hybrid B3PW91 with α=0.3. The results for the three aforementioned volumes of the compound are shown in Figs. 4 and 5. The Fermi level is set to zero, as denoted by the vertical dashed line.

The results, consistent with the band structure results, implies that the total and partial DOSs of $CeRhIn_5$ are not drastically changed by pressure. The total and partial DOSs are very similar for the three considered volumes of the compound, as can be seen from any rows of Fig. 4 or Fig. 5.

However, the DOSs, as discussed below, show that the degree of localization more significantly affects the partial and total DOSs of $CeRhIn_5$ than pressure. The results show that the 4f-Ce DOSs and as a result total DOSs around E_F are splitted by GGA+U in the three considered volumes. The GGA+U functional causes the occupied (unoccupied) total and 4f-Ce DOSs to shift towards lower (higher) energies than E_F with respect to the DOSs predicted by PBE-GGA. These shifts lead to a reduction of DOSs at the Fermi level. These results are in agreement with the previous calculations for other strongly correlated cases.[4,5] Similar to the GGA+U approach, the hybrid B3PW91 functional splits the occupied and unoccupied total and partial 4f-Ce DOSs compared to the PBE-GGA DOSs, see third and first rows of Fig. 4 or Fig. 5. However, the B3PW91 splitting is less than the GGA+U splitting. Thus, occupied and unoccupied states remain closer to the Fermi level after applying B3PW91 than GGA+U, see Figs. 4 and 5. This implies that the degree of localization is predicted to be less (more) by B3PW91 than by GGA+U (PBE-GGA), as discussed in our recent report[6]. The $DOS^{tot}(E_F)$ is affected by the degree of localization. In fact, like the effects of pressure on the value of the DOS at Fermi level, the total $DOS^{tot}(E_F)$ decreases as the degree of localization increases, but the effect of localization degree is more than the that of pressure. The value of total $DOS(E_F)$ is predicted to be the highest (lowest) by PBE-GGA (GGA+U) with the lowest (highest) degree of localization among the three considered functionals, see Table 2 as tabulated in the main manuscript.

Thus from the above observations in our theoretical study we can conclude that the pressure cannot but localization degree can considerably change the electronic structures of the strongly corelated $CeRhIn_5$ compound. We know that in experiment, however, the pressure can also remarkably change the properties of this system. This is so, because when we apply pressure experimentally, the localization of the system is also naturally changed in strongly correlated systems. But, in contrast to the experiment, when we apply pressure theoretically the degree of localization is not changed automatically. This is so, because in the current available theoretical approaches the degree of localization should be applied manually by selecting an appropriate functional. In other words, in the current theoretical schemes, the localization degree has not been related to the pressure because of the complicated nature of such a case-dependent relation. But, in experiment they are naturally related to each other. Therefore, in theory one should case by case consider a suitable degree of localization for every pressure[6].

Two Fortran programs are presented below based on the flowchart presented in Fig. 6. The names of these programs are max_con and and max_pf. They are used for extracting the maximum values of the thermoelectric parameters from the "CeRhIn5.condtens" file. This file is as an output of the BoltzTrap code. The BoltzTrap code itself uses some of the output files of the WIEN2k code as its inputs to produce "CeRhIn5.condtens" file. The "CeRhIn5.condtens" is an input file for max_con and and max_pf. The max_con program is written to find the maximum values of hole-like and electron-like of Seebeck coefficient, maximum values of electrical and thermal conductivities. The second program is used for finding the maximum values of the power factor and electronic figure of merit (Z) along a and c crystalline axes as well as the chemical potential and doping levels related to these maximums form "CeRhIn5.condtens file for $CeRhIn_5$ compound. The range of doping level in these programs is optional and can be controlled in max_con and max_pf programs.

3.1 Program max_con

This program calculates the maximum values of hole like Seebeck coefficient along the a axis (smaxxx_s), and along the c axis (smaxzz_s), electron like Seebeck coefficient along the a axis (sminxx_s), and along the c axis (sminzz_s), electrical conductivity along the a axis (sig_maxxx), electrical conductivity along the c axis (sig_maxzz), thermal conductivity along the a axis (kapp_maxxx), thermal conductivity along the c axis (kapp_maxzz).

```
character*20 :: title, filename, input_file
real*8, allocatable :: a(:,:)
real*8 Tmax, smaxxx_s, sminxx_s, smaxzz_s, sminzz_s,
     imaxxx_s, iminxx_s, imaxzz_s, iminzz_s,
sig_maxxx, sig_maxzz, imaxxx_sig, imaxzz_sig,
     kapp_maxxx, kapp_maxzz, imaxxx_kapp, imaxzz_kapp
integer b
```

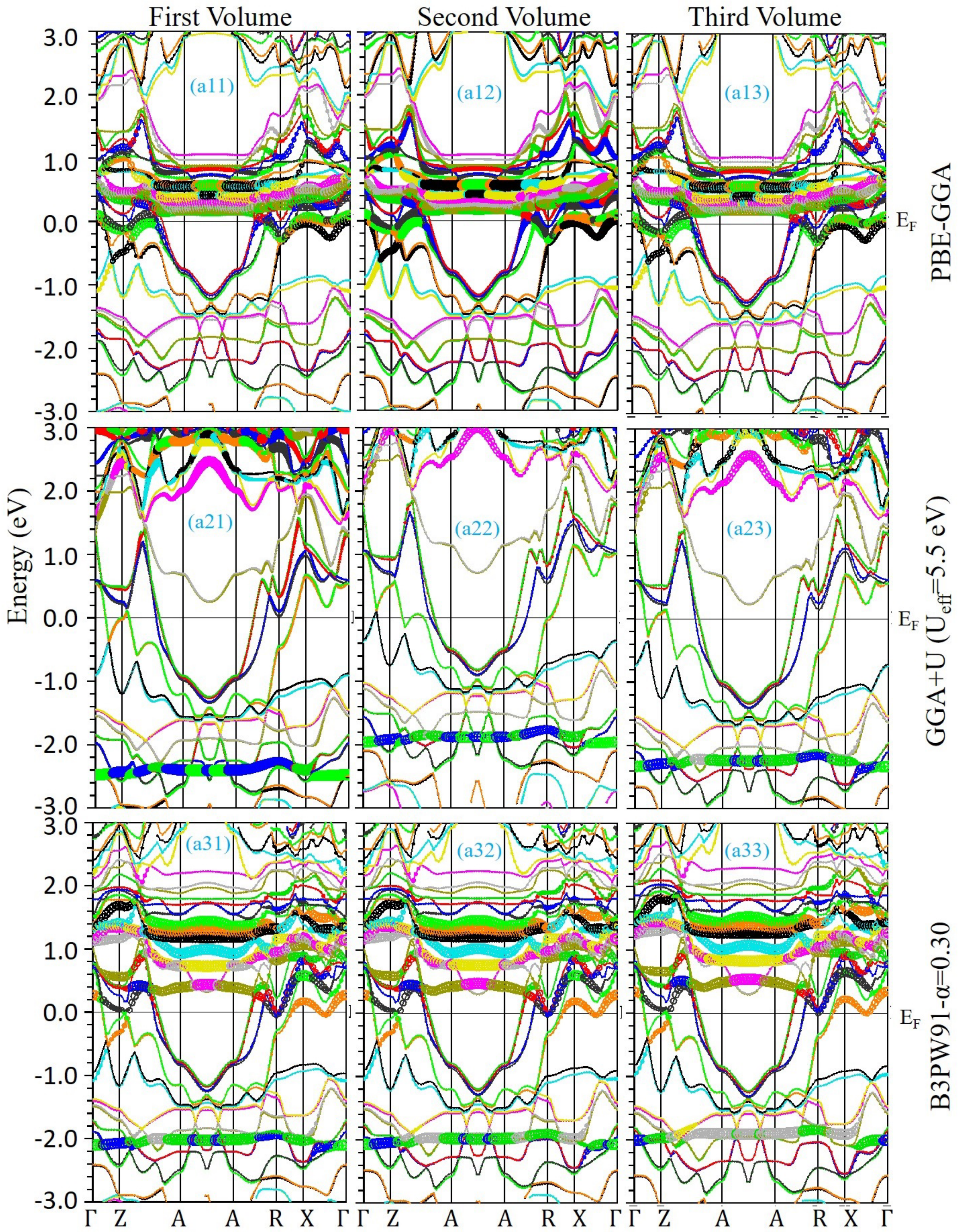

Fig. 2 Band structure of $CeRhIn_5$ for spin up direction utilizing a variety of XCFs in various volumes of $CeRhIn_5$ compound. The first volume is selected to be the experimental volume at zero pressure and the second (third) volume is 2% (5%) smaller than the first volume.

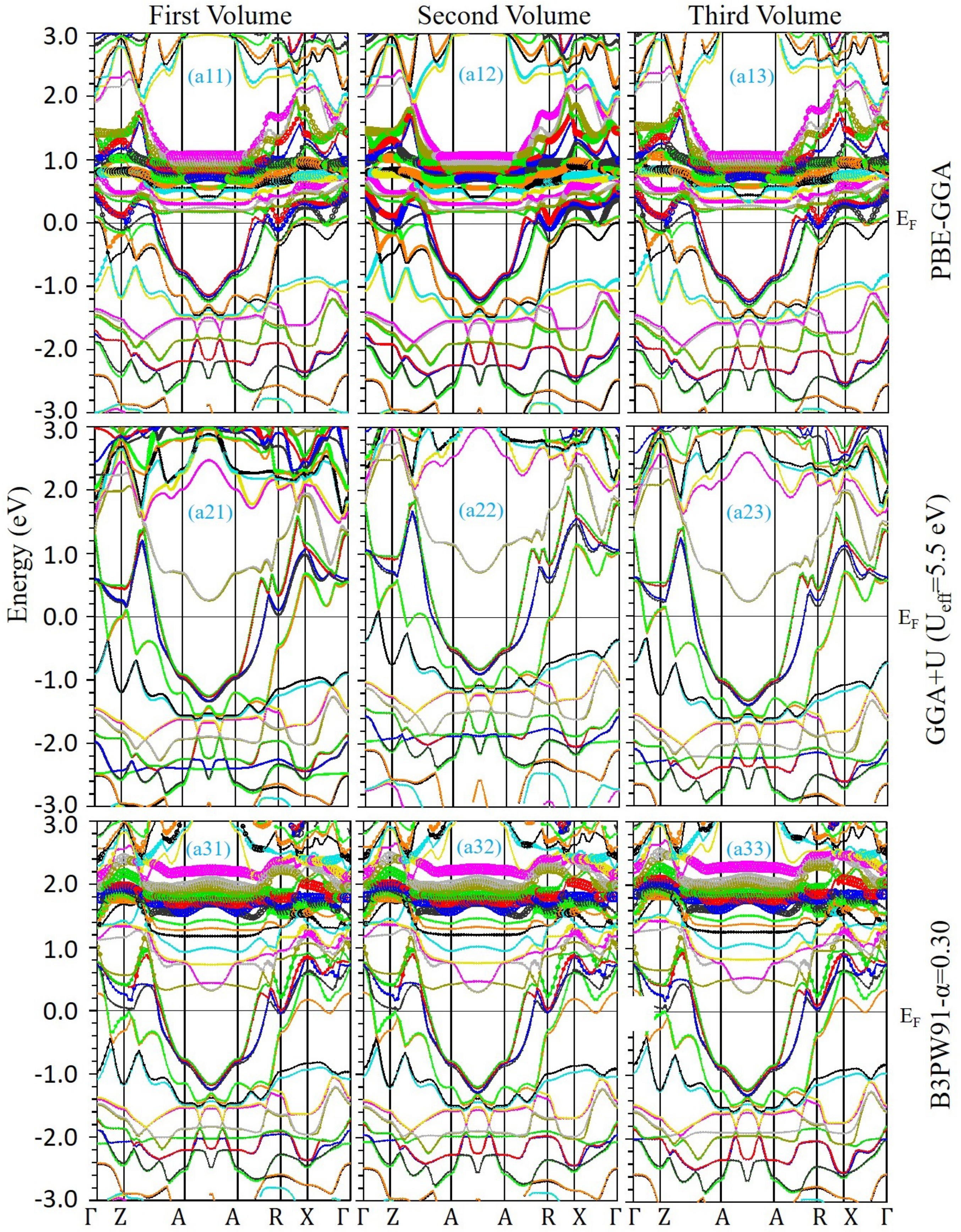

Fig. 3 Band structure of $CeRhIn_5$ for spin down direction utilizing a variety of XCFs in various volumes of $CeRhIn_5$ compound. The first volume is selected to be the experimental volume at zero pressure and the second (third) volume is 2% (5%) smaller than the first volume.

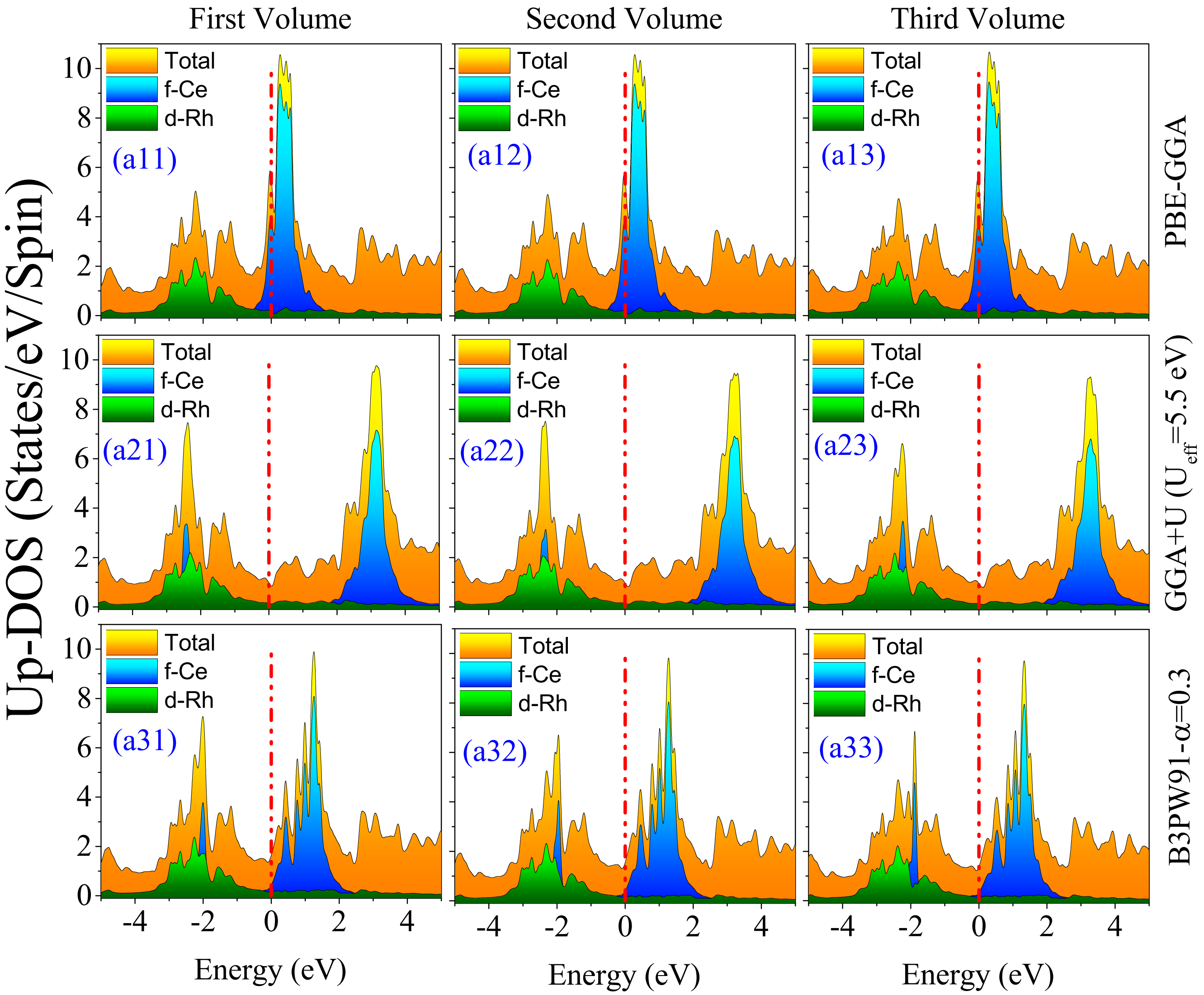

Fig. 4 Total as well as partial 4f-Ce and 4d-Rh DOSs of $CeRhIn_5$ for spin up using various XCFs for three different volumes of $CeRhIn_5$ compound. The first volume corresponds to the experimental volume at zero pressure. The second and third volumes are 2% and 5% smaller than the first volume, respectively.

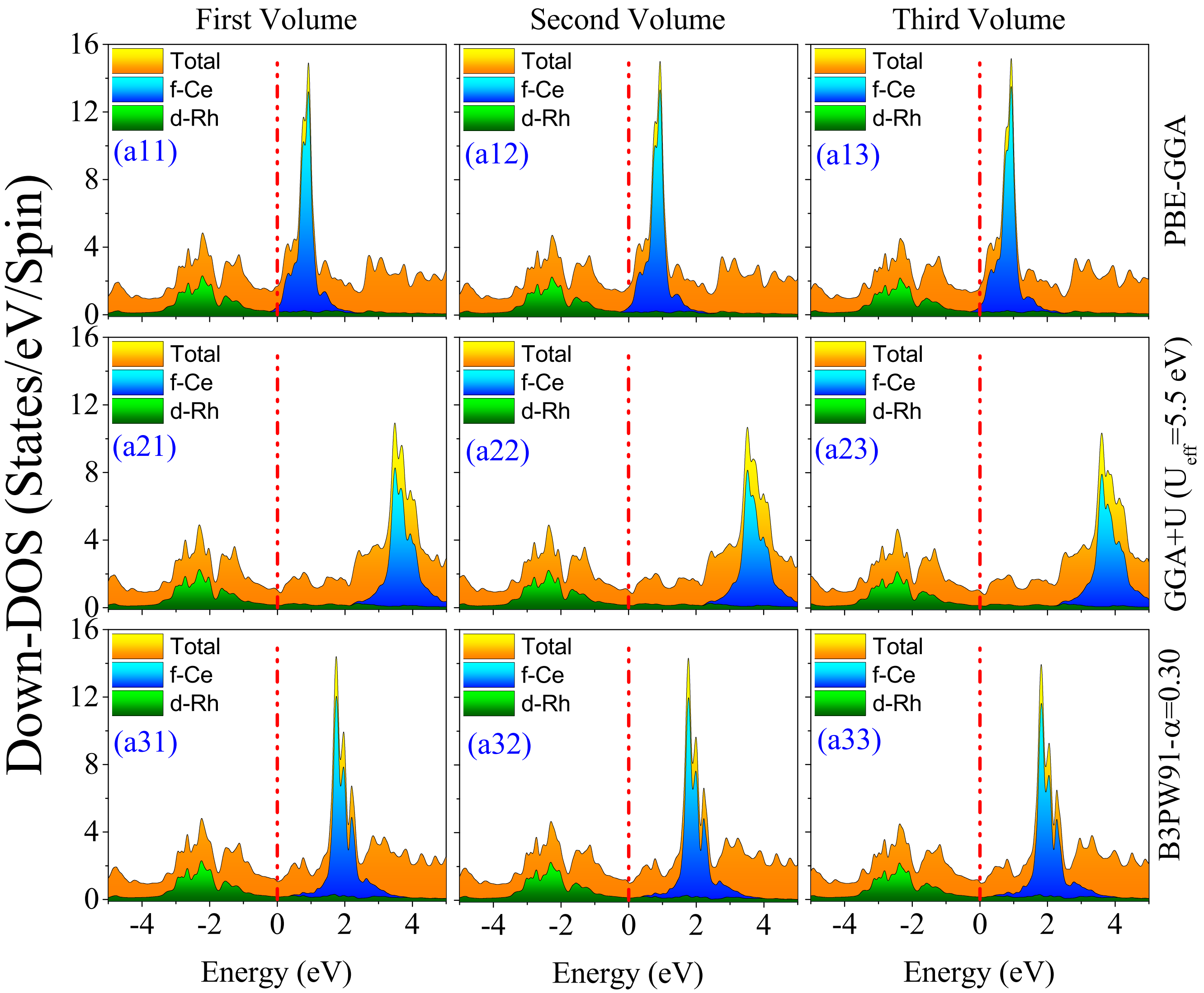

Fig. 5 Total as well as partial 4f-Ce and 4d-Rh DOSs of $CeRhIn_5$ for spin down using various XCFs for three different volumes of $CeRhIn_5$ compound. The first volume corresponds to the experimental volume at zero pressure. The second and third volumes are 2% and 5% smaller than the first volume, respectively.

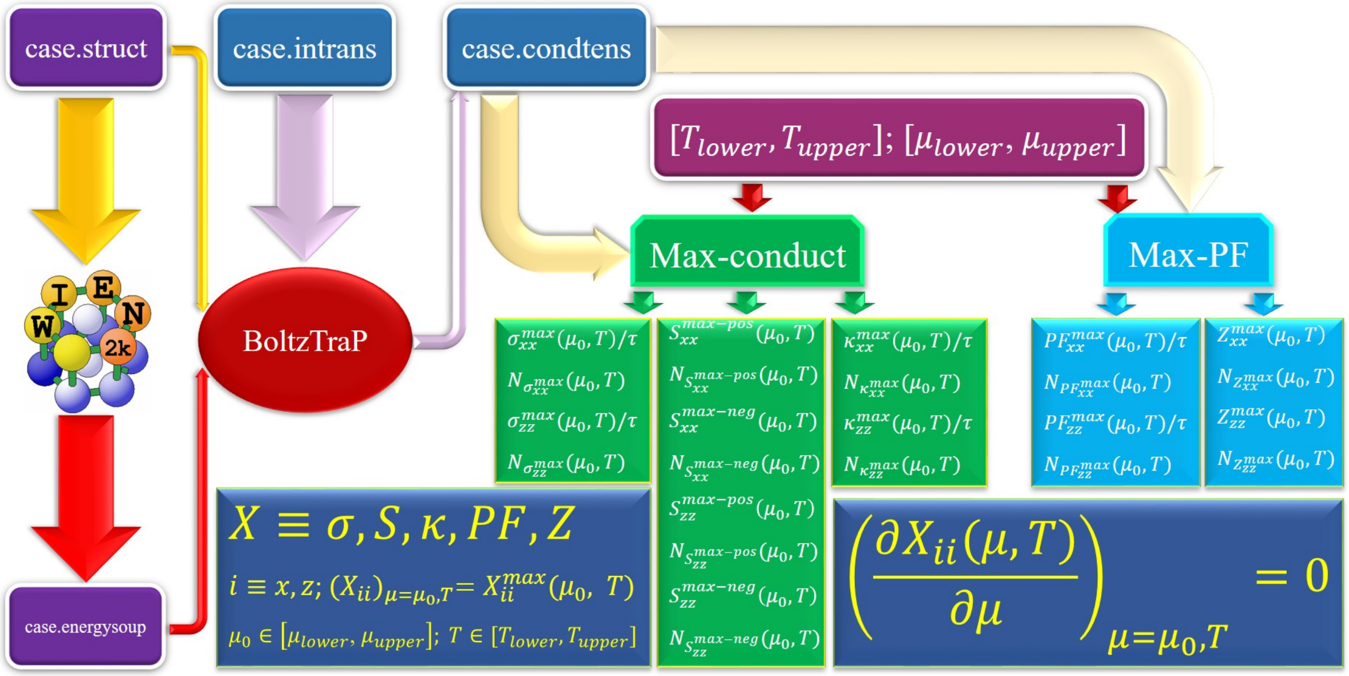

Fig. 6 Flowchart of the taks preformed to represent the thermoelectroc results reported in this work.

```
print*, "Intput file name?"
read*, filename
input_file = trim(filename) // '.condtens'
print*, 'Tmax?'
read*, Tmax
print*, "Minimun value for doping level (1/cm^3)?" !
    At this line the user determines the minimum of
doping level in carrier/cm^3 unit
read*, Doplev_min
print*, "Maximum value for doping level (1/cm^3)?" !
    At this line the user determines the maximum of
doping level carrier/cm^3 unit
read*, Doplev_max
print*, "Volume of system (a.u^3)?" !At this line the
     user determines the volume of system in a.u^3
    unit
read*, Vol
Vol=Vol*(0.148187e-24) ! the unit of volume is
    calculated in cm^3 unit
Doplev_min = Doplev_min*Vol ! the minimum of doping
    level is calculated in carrier/uc unit
Doplev_max = Doplev_max*Vol ! the maximum of doping
    level is calculated in carrier/uc unit
print*, Vol, Doplev_min, Doplev_max ! the volume in
    cm^3 unit, and the minimum and maximum of
doping level in carrier/uc are printed to check by
    user
open(2, file = input_file)
b = -1
do while (io /= -1)
      read(2,*, iostat=io)
      b=b+1
end do
print*, b
allocate (a(b,30))
close(2)
io = 0
open(3,file='output_s') ! Output file includes
    temperature, maximum values of hole and electron
    like
Seebeck coefficients along both a and c axes, the
    chemical potential and the doping level related
    to these
maximum values.
open(4,file = 'output_sig') ! Output file includes
    temperature, maximum values of electrical
conductivities along both a and c axes, the chemical
    potential and the doping level related to these
maximum values.
open(14, file = 'output_kap') ! Output file includes
    temperature, maximum values of thermal
conductivities along both a and c axes, the chemical
    potential and the doping level related to these
maximum values.

                  ti = 0
1000  ti = ti + 1
            smaxxx = -1
            imaxxx_s = 0
            smaxzz = 0
            imaxzz_s = 0
            sminxx = 0
            iminxx_s = 0
            sminzz = 0
            iminzz_s = 0
            sig_maxxx = 0
            imaxxx_sig = 0
            sig_maxzz = 0
            imaxzz_sig = 0
            kapp_maxxx = 0
            imaxxx_kapp = 0
            kapp_maxzz = 0
            imaxzz_kapp = 0
            io = 0
open(2, file=input_file)
```

```
read(2, *) title
do i = 1, b-1
      read (2, *)(a(i, j), j = 1, 30)
      if (a(i, 2) == ti) then
            if (a(i, 3) <= Doplev_max .And. a(i,3) >
                = Doplev_min) then !This line limits
                the doping levels of maximum
                thermoelectric parameters.
!#####################################################
!This section of program calculates the hole and
    electron like of Seebeck coefficient along the a
    axis, i.e., smaxxx and sminxx, as well as the row
     number of these values, i.e., imaxxx_s and
    iminxx_s, respectively at ti temperature.

if (a(i,13) > smaxxx) then
      smaxxx=a(i,13)
       imaxxx_s=i
end if
if (a(i,13)<sminxx) then
      sminxx=a(i,13)
    iminxx_s=i
end if
!#####################################################
!This section of program calculates the maximum
    values of hole and electron like of Seebeck
    coefficient along the c axis, i.e., smaxzz and
    sminzz, as well as the row number of these values
    , i.e., imaxzz_s and iminzz_s, respectively at ti
     temperature

if (a(i, 21) > smaxzz) then
      smaxzz = a(i, 21)
    imaxzz_s = i
end if
if (a(i, 21) < sminzz) then
      sminzz = a(i, 21)
    iminzz_s = i
end if
!#####################################################
!This section of program calculates the maximum value
     of electrical conductivity along the a (
    sig_maxxx) and c axes (sig_maxzz), as well as the
     row number of these values, i.e imaxxx_sig and
    imaxzz_sig, respectively at ti temperature
if (a(i, 4) > sig_maxxx) then
      sig_maxxx = a(i, 4)
      imaxxx_sig = i
end if
if (a(i,12) > sig_maxzz) then
      sig_maxzz = a(i,12)
      imaxzz_sig = i
end if
!#####################################################
!This section of program calculates the maximum value
     of thermal conductivity along the a (kapp_maxxx)
     and c axes (kapp_maxzz), as well as the row
    number of these values, i.e imaxxx_kapp and
    imaxzz_kapp, respectively at ti temperature
            if (a(i, 22) > kapp_maxxx) then
                  kapp_maxxx = a(i, 22)
                  imaxxx_kapp = i
            end if
            if (a(i, 30) > kapp_maxzz) then
                  kapp_maxzz = a(i,30)
                  imaxzz_kapp = i
            end if
      end if
end if
!#####################################################
end do
write(3,200) ti, a(imaxxx_s,1), a(imaxxx_s,3), a(
    imaxxx_s,13), a(iminxx_s,1), a(iminxx_s,3), a(
    iminxx_s,13), a(imaxzz_s,1), a(imaxzz_s,3), a(
    imaxzz_s,21), a(iminzz_s,1), a(iminzz_s,3), a(
    iminzz_s,21)
200   format(f10.4,4(1x, f10.4, 1x, f15.8, 1x, e15.8)
    )
       write(4,100) ti, a(imaxxx_sig,1), a(imaxxx_sig
           ,3), a(imaxxx_sig,4), a(imaxzz_sig,1), a(
           imaxzz_sig,3),    a(imaxzz_sig,12)
       write(14,100) ti, a(imaxxx_kapp,1), a(
           imaxxx_kapp,3), a(imaxxx_kapp,22), a(
           imaxzz_kapp,1), a(imaxzz_kapp,3), a(
           imaxzz_kapp,30)
100   format(f10.4,2(1x,f10.4,1x,f15.8,1x,e15.8))
            close (2)
            if (ti<Tmax) then
            goto 1000
      end if
\end
!#####################################################
```

3.2 Program max_pf

This program calculates the maximum values of power factor along the a (PF_maxxx) and c axes (PF_maxzz), the maximum values of electronic figure of merit along the a (ZT_maxxx) and c axes (ZT_maxzz)

```
      character*20 :: title, filename, input_file
      real*8, allocatable :: a(:,:), con(:,:), seeb
          (:,:),seeb2(:,:),PF(:,:),c(:,:),ZT(:,:), d
          (:,:)
      real*8 Tmax, PF_maxxx, PF_maxzz, imaxxx_PF,
          imaxzz_PF, ti, ZT_maxxx, ZT_maxzz,
          imaxxx_ZT,
      imaxzz_ZT, Doplev_min, Doplev_max, Vol
      integer b
      print*, "Input file name?"
      read*, filename
      input_file=trim(filename) // '.condtens'
      print*, 'Tmax?'
   read*, Tmax
      print*, "Minimun value for doping level (1/cm
          ^3)?"
      read*, Doplev_min
      rint*, "Maximum value for doping level (1/cm^3)
          ?"
      read*, Doplev_max
      print*, "Volume of system (a.u^3)?"
      read*, Vol
      Vol=Vol*(0.148187e-24)
      Doplev_min=Doplev_min*Vol
      Doplev_max=Doplev_max*Vol
      print*, Vol, Doplev_min, Doplev_max
      open(12, file=input_file)
      b=-1
      do while (io /= -1)
      read(12,*, iostat=io)
      b=b+1
      end do
      close(12)
      io=0
```

```
      print*, b
      allocate (a(b,30))
      allocate(seeb(3,3))
      allocate(con(3,3))
      allocate(seeb2(3,3))
      allocate(PF(b-1,6))
      allocate(ZT(b-1,6))
      open(12, file=input_file)
      open(13, file='con')
      open(14, file='seeb')
      open(15, file='seeb2')
      open(16, file='PF')
      open(17, file='ZT')
      read(12,*) title
      do i=1, b-1
      read (12,*)(a(i,j),j=1,30)
      m=4
      n=13
!####################################################
!This section claculates the square of Seebeck
    coefficent tensor (S^2).
      do k=1,3
            do l=1,3
                  con(k,l)=a(i, m)
                  Seeb(k,l)=a(i, n)
                  m=m+1
                  n=n+1
                  write(13,*) con(k, l)
                  write(14,*) seeb(k, l)
            end do
      end do

      do p=1,3
            do k=1,3
                  seeb2(p, k)=0
                  do l=1, 3
                        seeb2(p,k)=seeb2(p,k)+seeb(p,
                            l)*seeb(l,k)
                  end do
          write(15,*) seeb2(p,k)
            end do
!####################################################
! This section calculates the diagonal elements of
    power factor.
      PF(1, 1) = seeb2(1, 1)*con(1, 1) + seeb2(1, 2)*
          con(2, 1) + seeb2(1, 3)*con(3, 1)
      PF(1, 2) = seeb2(2, 1)*con(1, 2) + seeb2(2, 2)*
          con(2, 2) + seeb2(2, 3)*con(3, 2)
      PF(1, 3) = seeb2(3, 1)*con(1, 3) + seeb2(3, 2)*
          con(2, 3) + seeb2(3, 3)*con(3, 3)
      write(16,*) a(i,1),a(i,2),a(i,3), PF(1,1), PF
          (1,2),PF(1,3)
!####################################################
! This section calculates the diagonal elements of
    electronic figure of merit.
      ZT(1, 1) = PF(1, 1)/a(i, 22)
      ZT(1, 2) = PF(1, 2)/a(i, 26)
      ZT(1, 3) = PF(1, 3)/a(i, 30)
      write(17, *) a(i, 1), a(i, 2), a(i, 3), ZT(1,
          1), ZT(1, 2), ZT(1, 3)
      end do
      close(16)
      close (17)
!####################################################
! This section calculates the maximum values of power
     factor along the a (PF_maxxx) and c (PF_maxzz)
    axes at temperature ti.
      open(18, file = 'PF-max')
      open(19, file = 'ZT-max')
      ti=0
      allocate(c(b-1,6))
      allocate(d(b-1,6))
1000  ti=ti+1
            PF_maxxx = 0
            PF_maxzz = 0
            imaxxx_PF = 0
       imaxzz_PF = 0
       open(16, file = 'PF')
       do i=1, b-1
                  read(16,*) (c(i,j), j=1,6)
                  if (c(i,2) == ti) then
                        if (a(i, 3) <= Doplev_max .
                            And. a(i, 3) >=
                            Doplev_min) then
                              if (c(i,4)> PF_maxxx)
                                  then
                                    PF_maxxx = c(i,
                                        4)
                                    imaxxx_PF = i
                              end if
                              if (c(i, 6) > PF_maxzz)
                                   then
                                    PF_maxzz = c(i,
                                        6)
             imaxzz_PF=i
                              end if
                        end if
                  end if
            end do
!####################################################
! This section calculates the maximum values of
    electronic figure of merit along the a (ZT_maxxx)
     and c (ZT_maxzz) axes at temperature ti.
      ZT_maxxx = 0
      ZT_maxzz = 0
      imaxxx_ZT = 0
      imaxzz_ZT = 0
      open(17, file = 'ZT')
    do i=1, b-1
            read(17, *) (d(i, j), j = 1, 6)
            if (d(i, 2) == ti) then
                  if (a(i,3) <= Doplev_max .And. a(i
                      ,3) >= Doplev_min) then
                        if (d(i, 4) > ZT_maxxx) then
                              ZT_maxxx = d(i, 4)
                              imaxxx_ZT = i
                        end if
                        if (d(i, 6) > ZT_maxzz) then
                              ZT_maxzz = d(i, 6)
                  imaxzz_ZT = i
                        end if
                  end if
            end if
      end do
!####################################################
100   format (f10.4, 1x, f10.4, 1x,f10.4, e15.8, 1x,
    f10.4, 1x, f10.4, e15.8)
            write(18, 100) ti, c(imaxxx_PF, 1), c(
                imaxxx_PF, 3), PF_maxxx, c(imaxzz_PF,
                 1), c(imaxzz_PF, 3),
            PF_maxzz
      write(19, 100) ti, c(imaxxx_ZT, 1), c(imaxxx_ZT
          , 3), ZT_maxxx, c(imaxzz_ZT, 1),c(imaxzz_ZT
          , 3),
```

```
      ZT_maxzz
      close (16)
      close (17)
      if (ti<Tmax) then
                  goto 1000
      end if
      end
!#####################################################
```

References

1 R. T. Macaluso, J. Sarrao, P. Pagliuso, N. Moreno, R. Goodrich, D. Browne, F. R. Fronczek and J. Y. Chan, *Journal of Solid State Chemistry*, 2002, **166**, 245 – 250.

2 E. Moshopoulou, Z. Fisk, J. Sarrao and J. Thompson, *Journal of Solid State Chemistry*, 2001, **158**, 25 – 33.

3 E. Moshopoulou, J. Sarrao, P. Pagliuso, N. Moreno, J. Thompson, Z. Fisk and R. Ibberson, *Applied Physics A*, 2002, **74**, s895–s897.

4 M. Gamża, A. Źlebarski and J. Deniszczyk, *Journal of Physics: Condensed Matter*, 2008, **20**, 115202.

5 S. Jalali Asadabadi, *Phys. Rev. B*, 2007, **75**, 205130.

6 M. Yazdani-Kachoei, S. Jalali-Asadabadi, I. Ahmad and K. Zarringhalam, *Scientific Reports*, 2016, **6**, 31734.
